# User‐demand fast‐curable ocular glues enforced by multilength tunable networks

**DOI:** 10.1002/btm2.10323

**Published:** 2022-04-16

**Authors:** Hyeseon Lee, Ajeesh Chandrasekharan, Keum‐Yong Seong, Yeon Ji Jo, Samdae Park, Seonyeong An, Seungsoo Lee, Hyeji Kim, Hyungju Ahn, Sungbaek Seo, Jong Soo Lee, Seung Yun Yang

**Affiliations:** ^1^ Department of Biomaterials Science (BK21 Four Program) Life and Industry Convergence Institute, Pusan National University Miryang Republic of Korea; ^2^ Department of Ophthalmology Pusan National University College of Medicine and Medical Research Institute of Pusan National University Hospital Busan Republic of Korea; ^3^ SNvia Co., Ltd. Hyowon Industry‐Cooperation Building., Pusan National University Busan Republic of Korea; ^4^ Department of Polymer Science and Engineering Kyungpook National University Daegu Republic of Korea; ^5^ Industrial Technology Convergence Center Pohang Accelerator Laboratory, POSTECH Pohang Republic of Korea

**Keywords:** hyaluronic acid, hydrogel, ocular glue, photo‐crosslinking, tissue adhesive

## Abstract

Achieving fast and secure wound closure without ocular foreign body sensation is highly desired in ophthalmologic surgery. Sutureless approaches using tissue adhesives are gaining popularity, but their practical use is limited by the difficulty in controlling adhesion time and satisfying safety standards without compromising adhesive performance. Herein, we report user‐demand hydrogel‐forming ocular glues based on multilength photo‐crosslinkable hyaluronic acid (HA), achieving firm tissue adhesion under wet and dynamic conditions and possessing cornea‐like optical transparency. The HA‐based photocurable glue (HA photoglue) quickly seals wounds upon nontoxic low‐energy light exposure (320–500 nm, < 5 s, < 1 J cm^−2^), and its mechanical and adhesive properties are improved by introducing short and long crosslinkable moieties into HA through one‐step synthesis, forming multilength networks. Furthermore, the HA photoglue provides stable sealing in wet environments like ocular mucous surface, a clear vision with a light transmittance of more than 95% over the entire visible range, and a lubricating surface with minimal ocular sensation (generating less than 10% frictional force than suture groups). In a rabbit corneal incision model, the HA photoglue showed improved wound healing efficacy based on histological evaluation compared to control groups.

## 1 INTRODUCTION

Corneal injuries associated with anterior fluid outflow require an immediate watertight seal to reduce the risk of infection and leak‐related complications, such as hypotony, iris prolapse, corneal swelling, and lens dislocation.^1^, ^2^ Although clear corneal incisions inducing self‐sealing are commonly used in cataract surgeries (over 23 million surgeries annually worldwide), fast and reliable incision closure is necessary to reduce the potential risk of postoperative infections and improve postoperative visual acuity.^3^, ^4^ Ophthalmic suturing has been used as the gold standard for incision closure, but it is delicate and time‐consuming procedure that requires good technical skills. In addition, the sutured sites may lead to potential drawbacks, including leakage, secondary damage of ocular tissue, and infection.^5^, ^6^

Recently, sutureless approaches using glue‐type tissue adhesives have gained popularity as an alternative to corneal sealing and repair.^7^ Depending on the polymerization (or gelation) mechanism of liquid formulations, tissue glue can be classified into two types: physical or chemical adhesives.^8^ Physical adhesives involving polymer gelation by physical interactions, such as hydrogen bonding, ionic association, and molecular entanglements, form noncovalent and reversible crosslinks within the polymer network.^9^ Thus, they often exhibit low mechanical properties (break when the ocular blinking is applied), poor adhesive performance on wet tissues, and long gelation times. To secure a firm and reliable tissue adhesion, chemical adhesives based on reactive chemical polymerization or light‐triggered radical polymerization (or photo‐crosslinking) are mainly used in wound closure, including corneal sealing. For example, cyanoacrylate glue is widely used for ocular injuries and defects, due to its instant strong adhesion to a wet corneal surface.^10^ However, several complications associated with low biocompatibility, poor transparency, and difficulty in applying the glue to a target location due to rapid polymerization upon contact with any fluid have been reported.^4^, ^11^ Recently, in situ hydrogel‐forming adhesives (ReSure®) using reactive macromers (such as *N*‐hydroxylsuccinimide‐functionalized polyethylene glycol) and crosslinkers (such as trilysine acetate) were approved by the U.S. Food and Drug Administration (FDA) as ocular sealants to prevent leakage following cataract surgery with intraocular lens placement.^12^ This hydrogel adhesive is beneficial for providing a soft and lubricious surface barrier with increased biocompatibility; however, the uncontrollable gelation that is initiated immediately after mixing the reactive macromers and crosslinkers limit its use. These chemical tissue adhesives formed by reactive covalent crosslinking reactions are challenging to achieve user demands with respect to tissue adhesion and mechanical properties suitable for dynamic ocular environments (e.g., blinking).

Alternatively, hydrogel‐forming photo‐crosslinkable tissue adhesives composed of hydrophilic polymers functionalized with radical‐sensitive groups (namely, acrylate or methacrylate groups) and radical‐generating photoinitiators (PIs) have been gaining attention owing to their broad controllability in mechanical properties, curing kinetics, and adhesive performance depending on the crosslinking density. In addition, naturally derived biopolymers functionalized with photo‐crosslinkable groups are beneficial for wound healing after forming three‐dimensional hydrogel networks by photo‐crosslinking at the wound sites.^4^ For instance, methacrylate (MA)‐functionalized gelatin (GelMA)‐based tissue glue exhibited good adhesive and regenerative performance in a corneal injury model.^13^ However, GelMA hydrogel adhesives normally require long light exposure and high PI concentrations that could induce cytotoxicity during or after photo‐crosslinking,^14^ thus, potentially limiting their use in clinical applications. Although other natural polymers, including hyaluronic acid (HA), alginate, and chitosan, have also been used for ocular applications by the introduction of photo‐crosslinkable moieties (e.g., MA) to polymer chains,^13^, ^15^, ^16^, ^17^, ^18^, ^19^, ^20^, ^21^ photocuring conditions requiring long exposure to high‐energy light sources exceed the general guidelines for limiting light exposure to the eye (1 J cm^−2^ in 315–400 nm).^22^ In addition, the low substitution rate of photosensitive groups in hydrogel‐forming photocurable glues may result in poor tissue adhesion due to low mechanical strength and vulnerability to external stress.^23^

Tough adhesive hydrogels with multiscale networks showed good mechanical stability under applied force by the energy dissipation mechanism.^24^ Generally, multi‐network or double‐network hydrogels have been prepared through additional polymerization or crosslinking in existing hydrogel network.^25^, ^26^ One of the hydrogel networks provides material strength due to its tightly crosslinked structure, while the other loosely crosslinked structure could dissipate energy across the network through its flexible moieties. However, multiple preparation steps to form the tough adhesive limit their application in clinics, which require rapid application in emergency situations.

A suitable approach to overcome the limitations of existing ocular adhesives should (i) offer strong tissue adhesion on wet and dynamic mucosal surfaces, (ii) enable fast curing while meeting safety guidelines of light exposure to the eye, (iii) have proper mechanical properties withstanding cyclic external stress, (iv) be amendable to quick application (user‐demand adhesion), (v) provide soft and lubricous surface to minimize the ocular foreign sensation, (vi) be transparent without loss of visibility, and (vii) be biocompatible to promote wound healing process.

**FIGURE 1:**
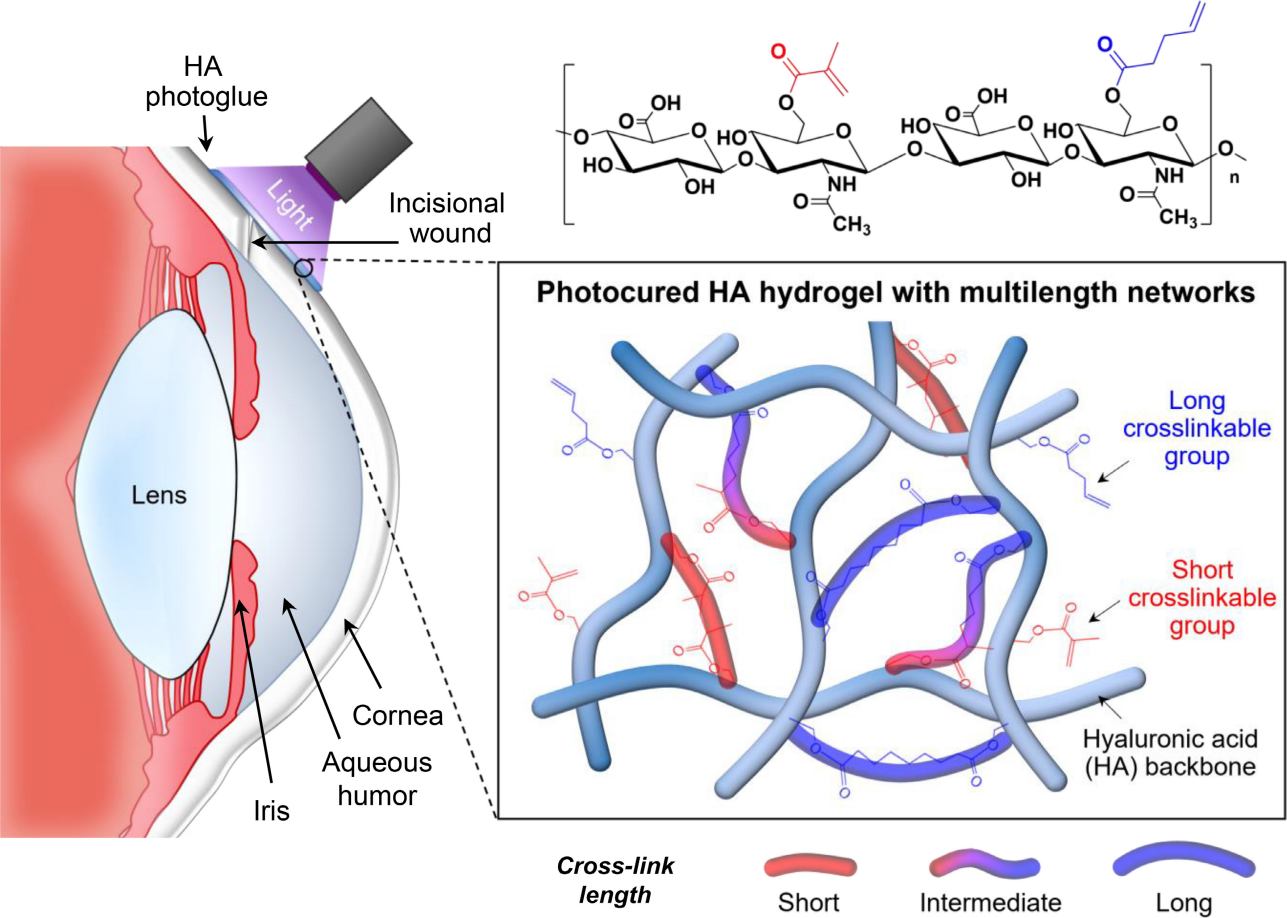
Schematic illustration showing the user‐demand hydrogel‐forming ocular glues based on copolymeric hyaluronic acids (HAs) with short and longphoto‐crosslinkable groups, achieving a firm adhesion on wet corneal surface and possessing cornea‐like optical transparency. The HA‐based photocurable glue (HA photoglue) offers a multilength‐networked, transparent, and watertight hydrogel barrier on the wound site following low‐energy light exposure

Here, we report a highly engineered photocurable ocular glue that provides a multilength‐networked, transparent, watertight hydrogel barrier on the wound site following low‐energy light exposure (less than 1 J cm^−2^). Hydrogel‐forming fast‐curable glue is designed to form a multilength network structure based on copolymeric HAs with short and long photo‐crosslinkable groups, enabling increased mechanical stability and tissue adherence during the wound healing process (Figure 1). By controlling the substitution ratio of short and relatively long photo‐crosslinkable moieties in HA polymer chains through one‐step synthesis, the mechanical and adhesive properties of the photocured hydrogels were optimized to improve their flexibility without decreasing the mechanical strength. The formulation of HA‐based photocurable glue (HA photoglue) consisting of photo‐crosslinkable HAs and PIs has been determined to improve its usability and safety for easy clinical application, without affecting its fast‐curing performance (less than 5 s). The long‐term stability and lubricity of HA photoglues under wet and dynamic conditions were evaluated through *in vitro* burst tests and *ex vivo* friction tests, respectively. The HA photoglue containing approximately 90% water after photo‐crosslinking showed excellent transparency with a light transmittance of more than 95% over the entire visible range. Finally, the wound healing efficacy and biological safety of the HA photoglue were assessed using a rabbit corneal incision model.

## 2 EXPERIMENTAL PROCEDURE

### 2.1 Materials

HA (Mw: 100 kDa, PDI: 2.01) was purchased from SNVia (Busan, Korea). Methacrylic anhydride (MAA), 4‐pentenoic anhydride (PEA), lithium phenyl‐2,4,6‐trimethylbenzoylphosphinate (LAP), sodium hydroxide (NaOH), porcine skin gelatin, ethanol, acetone, dimethyl sulfoxide, and brilliant blue FCF were purchased from Sigma‐Aldrich (Missouri, USA). Polydimethylsiloxane (PDMS, Sylgard 184) was purchased from Dow Corning (Michigan, USA).

### 2.2 Synthesis of photo‐crosslinkable polymers

To synthesize HA‐based photo‐crosslinkable polymers, HA (1.0 g) was dissolved in a mixture of 10 ml distilled (DI) water and 2 ml dimethyl sulfoxide. The HA solution was cooled to 5°C and the pH was adjusted to 8.0 using a 1 M NaOH solution. A mixture of five equivalents of MAA and PEA (4:1, 3.5:1.5, or 2.5:2.5) with respect to the disaccharide unit of HA was added dropwise over a period of 1 h. The pH was simultaneously maintained between 8.0 and 10.0 by adding 1 M NaOH solution. The temperature and pH were maintained for another 23 h, after which the reaction mixture was added slowly to a 94% ethanol and acetone mixture. The obtained precipitate was filtered and washed with 94% ethanol to give copolymeric HA with two photo‐crosslinkable groups (HAMA‐PA) as a white solid, which was stored at −20°C until use. Methacrylated HA (HAMA), used as a control, was prepared following a method reported previously.^23^ Briefly, 1 g of HA was dissolved in 10 ml of DI water and the pH was adjusted to 8.0, using 1 M NaOH at 5°C. Then, four equivalents of MAA, with respect to the disaccharide unit of HA, were added dropwise over a period of 30 min. The pH was simultaneously maintained between 8.0 and 10.0 by adding 1 M NaOH for another 23 h. The reaction mixture was then precipitated in 94% ethanol, and the solid was washed with 94% ethanol, frozen at −30°C, lyophilized, and stored at −20°C until use.

GelMA, used as a control, was prepared following a method reported previously.^23^ Briefly, 1.0 g of type A porcine skin gelatin was dissolved in 10 ml phosphate‐buffered saline (PBS) at 60°C and 2.58 g MAA was added, and the solution was stirred further for 4 h. The macromer solution was dialyzed against DI water for 1 week at 40°C with frequent changes in DI water to remove the salts and methacrylic acid. The solution was frozen at −55°C, lyophilized, and stored at −20°C until use. ^1^H nuclear magnetic resonance (NMR) spectra were obtained using a Bruker 600‐NMR spectrometer (Massachusetts, USA). This was used to confirm the functionalization of the photo‐crosslinkable methacrylate (MA) or 4‐pentenoate (PA) groups to calculate the total degree of substitution (*n* = 3).

### 2.3 Preparation of an HA‐based photocurable glue (HA photoglue)

The formulation of a HA photoglue was prepared by first dissolving 0.1% (w/v) LAP (PI) in DI water at 25°C followed by the addition of photo‐crosslinkable HA polymers with different substitution ratios of MA and PA (MA:PA = 10:0, 9:1, 7:3, and 5:5) to obtain 10% (w/w) solutions. The dynamic viscosity of HA photoglues with different polymer concentrations (5, 10, 15, and 20% (w/w)) was measured using a MicroVISC viscometer (RheoSence Inc., California, USA) at 24 ± 0.05°C. To measure the discharge force of liquid formulations, HA photoglues were filled in a 1 ml Luer‐Lok syringe (BD Korea, Seoul, Korea) with a 30‐gauge needle. The pre‐filled syringe was compressed using a universal testing machine at 100 mm min^−1^. For spreading tests, 20 μl of HA photoglues with different polymer concentrations (5, 10, 15, and 20% (w/w)) were dropped onto the porcine eyeball. Brilliant blue FCF was added to the HA photoglues to visualize the spreading area. Three repeats were examined for each test.

### 2.4 Cytotoxicity assay

Human conjunctival epithelial cells were obtained from the Biobank of Pusan National University Hospital and maintained in Medium 199 (Gibco, New York, USA) with 10% fetal bovine serum (Gibco, New York, USA) and 1% antibiotic–antimycotic solution (Sigma‐Aldrich, Missouri, USA). The cells were maintained at 37 °C and 5% carbon dioxide (CO_2_) in a humidified atmosphere with media changes every 2 or 3 days. The cytotoxicity of HA photoglues was evaluated by measuring the cell viability after exposure to different PI concentrations and light irradiation times. To perform the cell viability assay, the cells were seeded in 24‐well plates at a density of 2 × 10^4^ cells/well and incubated for 24 h. The first set of experiments was performed with different concentrations of LAP (0.01, 0.03, 0.05, 0.07, 0.1, 0.3, and 0.5% (w/v)) for 24 h. The second set of cell experiments was conducted after light exposure (5, 10, 20, 40, and 60 s) of 230 mW cm^−2^ light from the UV‐LED curing system (Omnicure S2000; Excelitas, Massachusetts, USA; Figure S1). The cells were supplemented with 10 μl of water‐soluble tetrazolium salt 1 (WST‐1) reagent (EZ‐Cytox; DogenBio, Seoul, Korea) and incubated for 1–2 h. Cell viability was measured based on the colorimetric optical density (OD) value at 450 nm using a microplate reader (AMR‐100; Allsheng Co., Ltd., Hangzhou, China). Cell images were obtained using inverted fluorescence microscope after Calcein‐acetoxymethylester (Calcein‐AM; Thermo Fischer, Massachusetts, USA) staining. Three repeats were examined for each group.

### 2.5 Rheological measurement

Rheological experiments were performed using a stress‐controlled rheometer (MCR 302, Anton Paar, Graz, Austria) equipped with a photocuring system (Omnicure S2000) and a temperature‐controlled bath. Radiation with an intensity of ~230 mW cm^−2^ was exposed through a transparent bottom‐plate fixture (*n* = 3). A 20 mm aluminum plate was used for the top geometry. Time‐sweep oscillatory tests were performed with plate‐plate geometry at ambient temperature (24–25°C), 1 Hz frequency, 0.4 mm gap, and 1% strain. To equilibrate and inhibit photo‐crosslinking by dissolved oxygen in the solution, a 1‐min delay to onset was processed.^27^ The gelation of HA photoglues was determined at the time point when the storage modulus (G') inversed the loss modulus (G'').

### 2.6 Mechanical properties of HA photoglues

HA photoglues were prepared by crosslinking the photocurable HA solutions filled in a dog bone PDMS mold with 1.5 mm thick under light irradiation (320–500 nm, ~230 mW cm^−2^) for 4.3 s. The light source was installed at a distance of 5 cm to irradiate the entire area of specimens. The mechanical properties of the photo‐crosslinked HA photoglues were measured using a mechanical tester (34sc‐1, Instron, Massachusetts, USA) in tensile mode at a strain rate of 1 mm min^−1^.^23^ The tensile strength and elongation were determined at the maximum point of stress and strain, respectively, in the stress–strain curves. The toughness was calculated from the area below the stress–strain curve until fracture. The elastic modulus was evaluated by obtaining the initial 5% of the slope in the strain–stress curves. Five repeats were examined for each group at room temperature (24–25°C).

### 2.7 Synchrotron‐based transmission small‐angle X‐ray scattering (TR‐SAXS) measurement

TR‐SAXS were performed at the 9A U‐SAXS beamline of the Pohang Accelerator Laboratory (PAL) in Korea. The experimental condition was set to a wavelength of 0.626 Å and sample‐to‐detector distance of 6.5 m. To avoid interference by X‐ray exposure to photo‐crosslinking in the hydrogel, fully crosslinked HA‐based hydrogels were prepared by light exposure for 120 s. The hydrogels of HA photoglues were exposed to an X‐ray beam for 10 s under atmospheric conditions without a pre‐treatment process. Scattered photons, induced by the homogeneity of HA hydrogels, were collected using a 2D charge‐coupled detector (MX170‐HS; Rayonix Ltd., Illinois, USA).

### 2.8 *In vitro* wound closure test

The adhesive bond strength of the HA photoglues was measured by a wound closure test.^23^ Corneal tissue (5 × 15 mm) was fixed between glass slides leaving a 6 mm section. The tissue was cut apart using a razor blade, and 40 μl of HA photoglues was dropped onto the corneal tissue. After photo‐crosslinking, the slides were placed on a mechanical tester for shear testing by tensile loading at a strain rate of 1 mm min^−1^. The shear strength was obtained at the maximum stress point. Five repeats were examined for each group at ambient temperature (24–25°C).

### 2.9 *Ex vivo* burst pressure test

For the intraocular pressure (IOP) test, a 5‐mm corneal incision was created on the explanted porcine cornea (*n* = 5). HAMA‐PA or HAMA solutions (20 μl) were applied at the incision site, followed by curing to seal the incision. Then, an 18‐gauge syringe needle connected to a peristaltic pump was inserted into the enucleated porcine eye globes, and PBS was pumped into the sealed porcine eye at a flow rate of 2 ml min^−1^ until leakage. The IOP was recorded using a water pressure sensor (SMC, Tokyo, Japan).

Burst pressure testing using HA photoglues was conducted using a standard method (ASTM F2392‐04) as described in the literature.^13^ In brief, a hole was created in the collagen substrate (4 × 4 cm) using a 3 mm diameter biopsy punch. Then, 20 μl of HA photoglues were dropped on the surface of the hole and the hole was immediately sealed upon light exposure. After fixing the tissue in the measuring apparatus connected to the peristaltic pump, the PBS solution was pumped at a flow rate of 2 ml min^−1^ until leakage. The peak pressure before the pressure loss was considered as the burst pressure. Five repeats were examined for each group.

### 2.10 *Ex vivo* friction test

The friction test to evaluate mechanical sensation by a corneal foreign body was performed after treatment with HA photoglues (20 μl) and sutures (Black silk 7–0, Ailee, Korea) onto the porcine cornea. The HA photoglues and sutures (3 or 7 stitches) were placed at the center of explanted corneal tissue (1 cm × 1 cm) fixed on a glass slide. The explanted eyelid was fixed on another glass slide and placed over the cornea. The friction force between the treated cornea and eyelid adhered on glass slides was monitored during repetitive movements (100 cycles) that mimicked the blinking eye at room temperature (24–25°C).

### 2.11 Optical properties of HA photoglues

The transmittance of the photo‐crosslinked HA photoglues was measured using a UV–Vis spectrometer (Libra S70; Biochrom, Cambridge, UK) at a wavelength of 200–800 nm. The sample was prepared by filling solutions with a thickness of 500 μm, followed by photo‐crosslinking as described above. The refractive index (RI) of the HA hydrogels was measured using an ellipsometer (RC2‐XF; J.A. Woollam, Nebraska, USA). Three repeats were examined for each group.

### 2.12 Efficacy test of HA photoglues with *in vivo* corneal incision model

The Pusan National University Hospital‐Institutional Animal Care and Use Committee reviewed and approved the animal protocol based on ethical procedures for scientific care (Approval Number PNUH‐2020‐162). Male New Zealand White rabbits (14–16 weeks, 3.0 ± 0.5 kg) were anesthetized by intramuscular injection of ketamine (35 mg/kg) and xylazine (10 mg/kg). Proparacaine 0.5% ophthalmic solution was dropped for topical anesthesia of the cornea. A 5‐mm full‐thickness corneal incision was induced until leakage occurred from the anterior chamber using a 2.8‐mm keratome. Seidel test and visual inspection using slit‐lamp biomicroscopy were performed on the rabbit eyes at 2, 7, and 14 days under anesthesia. The rabbits were then euthanized and the corneal tissue was explanted for histological analysis (*n* = 3 for each group).

### 2.13 Histopathological assessment

For histopathological analysis, the corneal tissues were corrected and fixed in 10% buffered formalin for at least 72 h. The fixed tissues were then embedded in paraffin and 4 μm sections were stained with hematoxylin and eosin (H&E) and toluidine blue, following standard procedures for histological analysis under a light microscope (Eclipse TS100; Nikon, Tokyo, Japan).

## 3 RESULTS

### 3.1 One‐step synthesis of HAs functionalized with multilength photo‐crosslinkable groups

**FIGURE 2:**
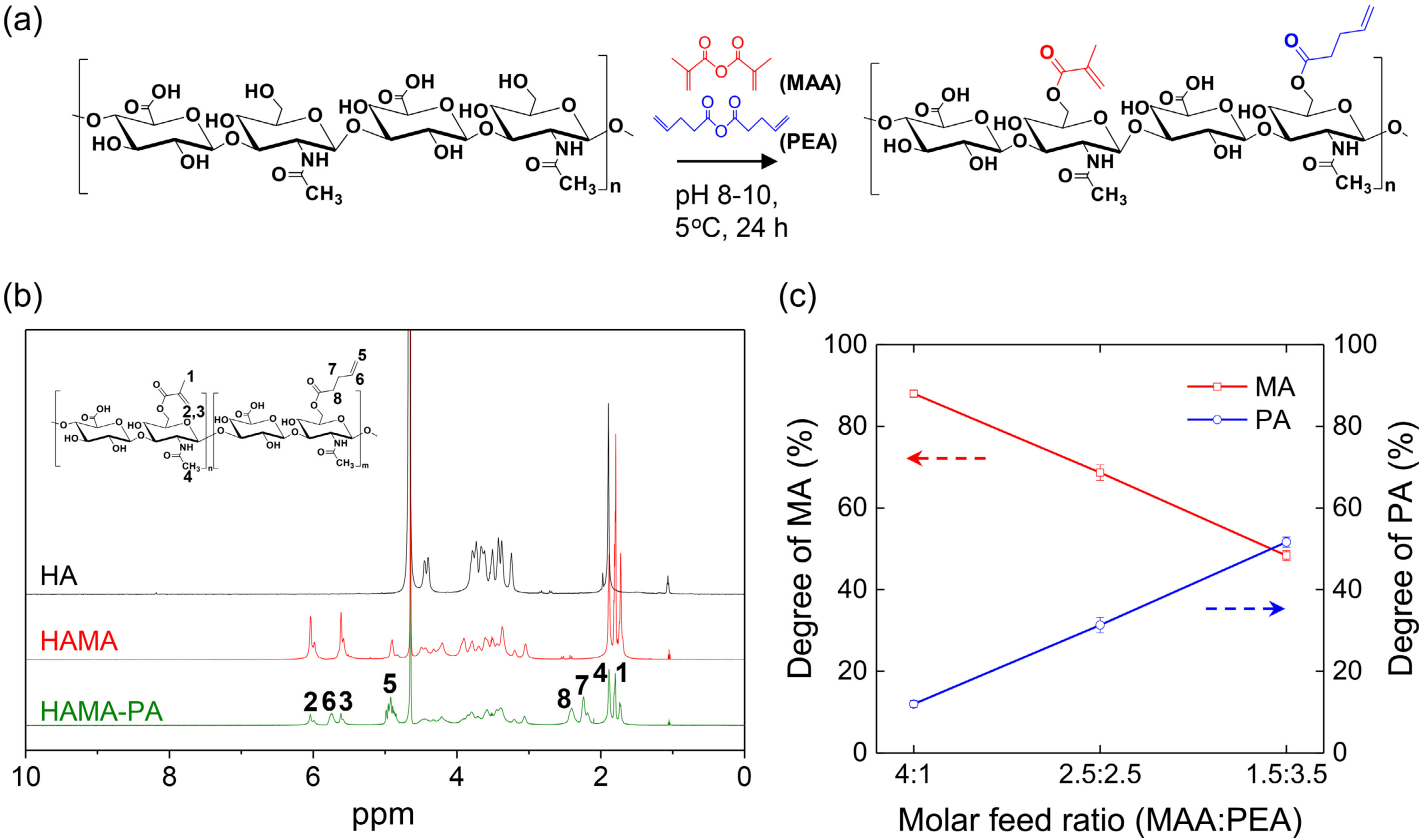
(a) Schematic for the one‐step synthesis of copolymeric hyaluronic acids (HAs) with multilength photo‐crosslinkable groups. (b) Representative ^1^H NMR spectrum of HA and its derivatives with photo‐crosslinking functionalities (methacrylated HA (HAMA) and copolymeric HA with two photo‐crosslinkable groups (HAMA‐PA) with methacrylate (MA):4‐pentenoate (PA) molar ratio of 7:3. Peaks corresponds to methacrylate protons (2, 3), pentenoate alkene protons (5, 6), methylene proton (7, 8), and methyl protons (4) in HA polymer chains. (c) Substitution ratio of MA and PA in the photo‐crosslinkable HAMA‐PAs prepared by different molar feed ratios of methacrylic anhydride:4‐pentenoic anhydride (MAA:PEA) (*n* = 3). Values and error bars represent the mean and the standard deviation

To obtain a fast‐curable hydrogel glues with multilength networks, HA was functionalized with short and relatively longer photo‐crosslinkable groups, MA and PA, respectively, by one‐step synthesis approach under esterification reaction conditions (Figure 2a). After the synthesis of photo‐crosslinkable HA derivatives, ^1^H NMR experiments were performed to characterize the functionalization of the photo‐crosslinkable groups and their degree of substitution (Figure 2b). Peaks at 6.18 and 5.76 ppm, and at 5.88, 5.07, 2.55, and 2.38 ppm confirmed the substitution of MA and PA groups, respectively. The HAMA was prepared as a control to compare the mechanical and adhesive properties of HAMA‐PA. By tuning the molar feed ratios of MAA and PEA, the substitution ratios of MA and PA in the HAMA‐PA polymer chains were precisely controlled (Figure 2c). PA was chosen as the relatively longer photo‐crosslinkable group because the precursor (PEA) was commercially available and PA‐functionalized HA (HAPA) showed good biocompatibility *in vivo*.^19^, ^28^ Since PEA with a relatively longer alkyl chain length is less soluble in aqueous reaction medium (especially at low temperature), a higher equivalent of PEA was required to obtain a similar degree of substitution between the MA and PA groups. Therefore, from the feed ratio of MAA:PEA of 4.0:1.0, 2.5:2.5, and 1.5:3.5, the copolymeric HAMA‐PAs having a substitution ratio of MA:PA of 9:1, 7:3, and 5:5, respectively, were obtained. Remarkably, the total degree of substitution of HA polymer chains for all feed ratios was found to be more than 200%, as confirmed by ^1^H NMR analysis, which is suitable for fast photo‐crosslinking reaction, although the total degree of substitution decreased with the increasing molar concentration of PEA (Figure S2 and Table S1). This one‐step synthetic approach to obtain multilength photo‐crosslinkable HA reduces the number of synthetic steps and improves the overall process efficiency, while minimizing time and cost.

### 3.2 Formulation of a HA photoglues

**FIGURE 3:**
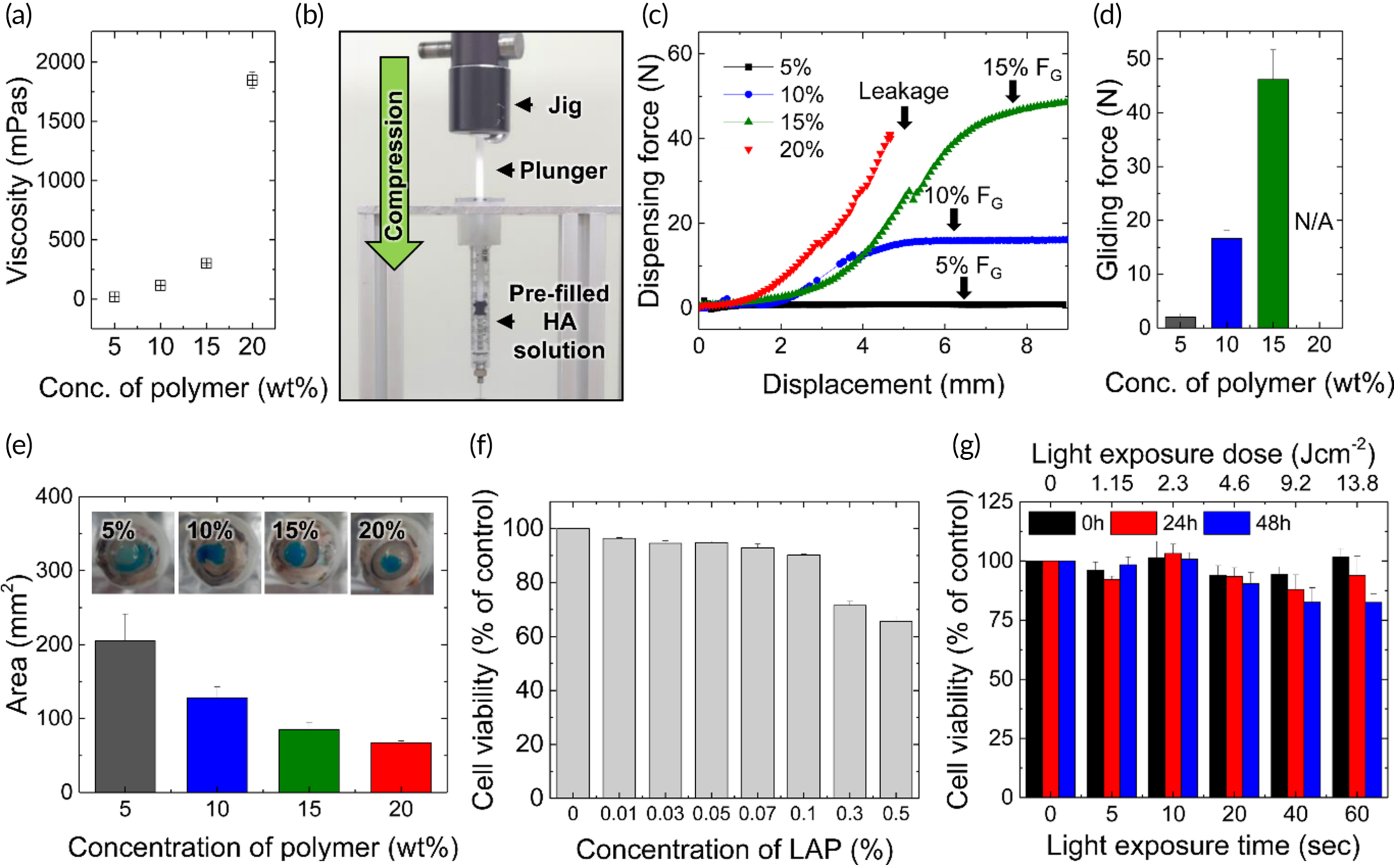
(a) Viscosity of aqueous HAMA‐PA solutions plotted as a function of polymer concentrations (*n* = 3). (b) Photograph showing the experimental set‐up for measuring the dispensing force of the HAMA‐PA solution filled in a syringe with a 30‐gauge needle. (c) Representative force‐displacement profile obtained during the compressing of syringe plunger to dispense HAMA‐PA solutions of different concentrations. (d) Average gliding force obtained from the force‐displacement tests (*n* = 3). (e) Spreading tests of HAMA‐PA solutions (20 μl) dispensed on the porcine eye. Blue dye was added in the HAMA‐PA solutions for visualization. (*n* = 3) (F, G) Cytotoxic effects of (F) photoinitiator (PI, LAP) concentrations (0, 0.01, 0.03, 0.05, 0.07, 0.1, 0.3, 0.5% (w/v)) and (g) light exposure time with intensity of 230 mW cm^−2^ (0, 5, 10, 20, 40, 60 s) on the human conjunctival epithelial cells (*n* = 3). Values and error bars represent the mean and the standard deviation

To determine the composition of HA photoglues for ocular applications, we first examined the viscosity of the HAMA‐PA solutions depending on the polymer concentration. Because the viscosity of a liquid glue is related to the dispensing pressure, an appropriate viscosity that can control the dispensing volume is desirable to improve usability. The viscosity of an aqueous HAMA‐PA with (molar ratio of 7:3 MA/PA) solution increased exponentially with increasing polymer concentration, resulting in 18.09 ± 0.05, 114.93 ± 0.51, 302.66 ± 16.62, and 1847.66 ± 68.25 mPa s for 5, 10, 15, and 20% (w/w) solutions, respectively (Figure 3a). The molar ratio between MA and PA in HAMA‐PA did not affect the viscosity of the aqueous HAMA‐PA solutions (Figure S3). The force to dispense the HAMA‐PA solution filled into the syringe with a 30‐gauge needle (commonly used in ophthalmology) was measured in compression mode, as shown in Figure 3b. As the polymer concentration in the syringe increased, the compressive force required to reach the plateau region, known as the gliding force (*F*_G_),^29^ enabling stable dispensing of the HAMA‐PA solution with minimal fluctuation increased (Figure 3c, d). The 20% (w/w) polymer solution was not discharged from the syringe at a force below 100 N. The 10% (w/w) HAMA‐PA solution was smoothly pressed out in seconds without a high break loose peak, observed in a lower viscous solution (5% (w/w)) (Figure S4).

As tissue glue is desirable for application in the target area, we investigated the spreading area of HAMA‐PA solutions (20 μl) dispensed on the extracted porcine eye. For this experiment, the porcine eyeball was fixed on a glass bottle, and the water‐soluble blue dye was added to the HAMA‐PA solutions for easy visualization. While 5% (w/w) HAMA‐PA solution showed a quick spread over the whole eye surface, higher concentration solutions of HAMA‐PA showed limited spreading on the convex cornea, indicating controllable application to the target area (Figure 3e). Considering usability and controllable covering on the ocular surface, 10% (w/w) HAMA‐PA solutions were selected to evaluate the adhesive performance of HA photoglues.

The safety of HA photoglues, including the photocuring process, is the most important factor for translation into the clinic, especially applications to the ocular surface. We considered two parameters (PI concentration and light dose) to evaluate the cytotoxic effects of the HA photoglue. For this purpose, cell viability tests were performed using human conjunctival epithelial cells, depending on the PI concentration and total light exposure (light dose: light intensity × exposure time). At a concentration of less than 0.1% (w/v) PI, the cytotoxic effect was not significant, resulting in a cell viability of more than 90% compared to the non‐treated group (Figure 3f). Similar results were observed in cytotoxicity tests using corneal stromal cells (Figure S5). As shown in Figure 3g, light exposure (320–500 nm) with an intensity of 230 mW cm^−2^ did not significantly affect the viability of human conjunctival epithelial cells after 24 h of incubation, but the long exposure time (60 s) reduced the cell viability after 48 h of incubation, indicating potential cytotoxicity by light exposure. Given the guidelines on the limit of light exposure in the near‐ultraviolet spectral region (315–400 nm),^22^ the total light dose to the eye should not exceed 1 J cm^−2^. Based on this criterion, a PI concentration of 0.1% (w/v) and a light exposure of 230 mW cm^−2^ for 4.3 s (0.98 J cm^−2^) were selected for developing a nontoxic photocurable tissue glue.

### 3.3 Rheological analysis and mechanical properties of HA photoglues

**FIGURE 4:**
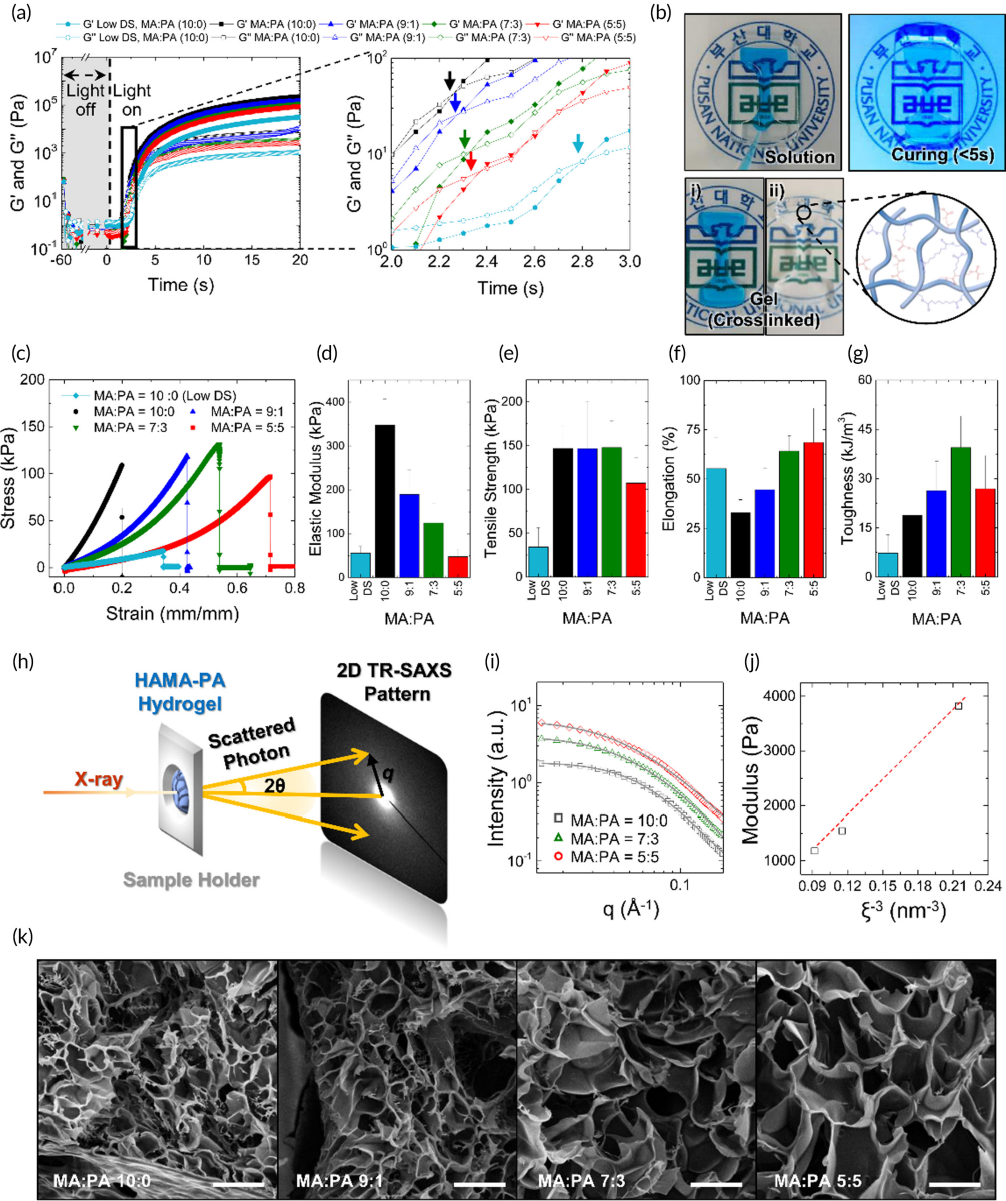
(a) Comparison of the G' and G" profiles of HA photoglues prepared using HAMA‐PA solutions with different molar ratios of MA and PA under 230 mW cm^−2^ light intensity as a function of exposure time. The gel points (crossover of G' and G") of each sample are indicated by arrows. (b) Photographs showing in situ hydrogel formation of HA photoglues. (Top) HA photoglue filled in a dog bone PDMS mold before (left) and after (right) light exposure. Blue dye was added into the glue solution for better visualization. (i) hydrogel with blue dye, (ii) neat hydrogel without dye. (c–g) Tensile tests of photo‐crosslinked hydrogels prepared from 10% (w/w) HA photoglues with different MA:PA ratios (*n* = 5). (c) Representative stress–strain curves, (d) elastic modulus, (e) ultimate tensile strength, (f) elongation, and (g) toughness. (h–j) X‐ray measurement of HA photoglues with different molar ratios of MA and PA (10:0, 7:3, 5:5). (h) Schematic illustrating the experimental set‐up of transmission small‐angle X‐ray scattering (TR‐SAXS). (i) SAXS intensity profiles (symbols) with correlation length model fitting results (solid lines). (j) Shear modulus (G') plotted as function of ξ^−3^. (k) Cross‐sectional scanning electron microscope (SEM) images of freeze‐dried hydrogels prepared by HA photoglues with different MA:PA ratios. Values and error bars represent the mean and the standard deviation

The gelation kinetics of HA photoglues were studied through a time‐sweep oscillatory test with plate–plate geometry. By applying small‐amplitude oscillatory shear, the formation of crosslinks and changes in the material properties, such as elastic (G') and viscous (G'') moduli, under light exposure (320–500 nm), were monitored with minimal disruptions to the chemical reaction.^30^ The evolution of G' and G" for HA photoglues with different molar ratios of MA and PA was measured under light exposure with 230 mW cm^−2^ and 1 rad^−1^ oscillation. As shown in Figure 4a, G' surpassed G" in 2.5 s for all testing solutions, indicating a polymer network (gel state) formed by photo‐crosslinking. This result demonstrated that the HA photoglues with a high substitution rate (>200%) enables rapid photocuring to achieve instant tissue adhesion under safe photo‐crosslinking conditions (less than 0.1% (w/v) PI and 1 J cm^−2^ light dose).

To investigate the mechanical properties of the HA photoglue after photo‐crosslinking, hydrogel‐type testing specimens were prepared using a dog‐bone‐shaped mold (Figure 4b). The representative stress–strain curves of the photo‐crosslinked hydrogels of HA photoglues obtained in the tensile tests are shown in Figure 4c. With increasing PA substitution rate in HA, the modulus of the photo‐crosslinked hydrogels gradually decreased from 350 kPa for HAMA to 50 kPa for HAMA‐PA (5:5) (Figure 4d). However, the tensile strength of the hydrogels at fracture did not significantly change with an increase of the PA substitution in the HA polymer chains (Figure 4e). Interestingly, the HAMA‐PA (7:3) hydrogel exhibited increased tensile elongation prior to breaking (60% elongation above the initial length) (Figure 4f); thus, it showed 110% higher toughness compared to HAMA without PA introduction (Figure 4g). This enhanced resistance against mechanical deformation would be critical to maintain its mechanical integrity under dynamic conditions, such as bending motion in the body (Figures S6 and S7). Compared to commercially available HAMA with a low DS (~60%) of MA, the HAMA‐PA (7:3) hydrogel exhibited significantly higher tensile strength (4.3‐fold) and toughness (5.4‐fold). In general, crosslinkers with shorter crosslink lengths provide higher tensile strength with reduced elongation; reverse results are observed for crosslinkers with longer crosslink lengths.^31^, ^32^ Interestingly, HAMA‐PA with multilength photo‐crosslinkable groups exhibited enhanced flexibility and elongation without decreasing mechanical strength, possibly because of the multilength networks in the hydrogels.

TR‐SAXS experiments were conducted to examine the internal structure of the HA photoglues. After photo‐crosslinking for 120 s to reach a fully crosslinked state, the testing hydrogels were mounted in the sample cell and measured under atmospheric conditions, as shown in Figure 4h. Meanwhile, Figure 4i presents a double logarithmic plot of circular averaged scattering intensity (*I*(*q*)), extracted from 2D TR‐SAXS patterns, as a function of the scattering vector *q*. Here, the scattering vector can be expressed by Equation (1)).

(1) $$q=\frac{4\pi \;\sin\left(\theta \right)}{\lambda }$$

where λ is the wavelength of the monochromated X‐ray beam, and 2*θ* is the scattering angle.

In Figure 4i, the experimental scattering intensity profiles for all HAMA‐PA hydrogels, indicated by scattered symbols, consistently presented the crossover point of the slope change, due to inhomogeneity (or density fluctuation) at the sub‐nanometer scale. To extract the microstructural information of the HAMA‐PA hydrogels, the scattering intensity (*I*(*q*)) profiles were fitted using the correlation length model in Equation (2).^33^

(2) $$I\left(q\right)=\frac{I\left(0\right)}{1+{\left(\mathrm{\xi q}\right)}^{n}}+B$$

where *I*(0), *B*, and *ξ* are the scattering intensities extrapolated to the scattering vector *q* = 0, background scattering, and correlation length, respectively. All scattering intensity profiles for the HAMA‐PA hydrogels were well‐fitted to the correlation length model, as indicated by the solid lines in Figure 4i. The correlation length (ξ) of crosslinked domains, extracted from the correlation length model fit, increased with increasing substitution ratio of longer PA groups (Figure 4i and Table S2). This result suggests that the introduction of photo‐crosslinkable PA groups in HA chains manipulates the length between crosslinks (free polymer chains) in the networks. In addition, the power‐law exponent *n* ~ 2 was consistently observed over the entire hydrogel, which is a general value for polymeric gel systems.^34^

According to the theory of rubber elasticity for a network of flexible chains, the shear modulus (G') is given by Equation (3)) with respect to ξ.^33^, ^35^

(3) $$G^{\prime }\propto \frac{\mathrm{kT}}{\xi^{3}}$$

where *k* and *T* are the Boltzmann constant and absolute temperature, respectively. As shown in Figure 4j, the shear modulus (G') at curing conditions (4.3 s) measured by the rheological analysis has a linear relationship with ξ^−3^ obtained from the correlation length model in Figure 4i. To observe the internal structure of the HA hydrogels after photocuring, the cross‐section of the freeze‐dried HA photoglues was characterized using scanning electron microscopy (SEM) (Figure 4k). The water pocket size (pore size) in the networks increased with increasing substitution degree of PA groups in the HA chains. HAMA‐PA (7:3) has two times larger pore size (26.65 ± 8.82 μm) compared to that of HAMA (14.23 ± 7.79 μm). Since the porous structure of the hydrogel applied to injured tissue is advantageous for the transport of therapeutic agents and oxygen, HAMA‐PA with higher porosity would be beneficial for wound healing.^36^

### 3.4 Adhesive performance of HA photoglues

**FIGURE 5:**
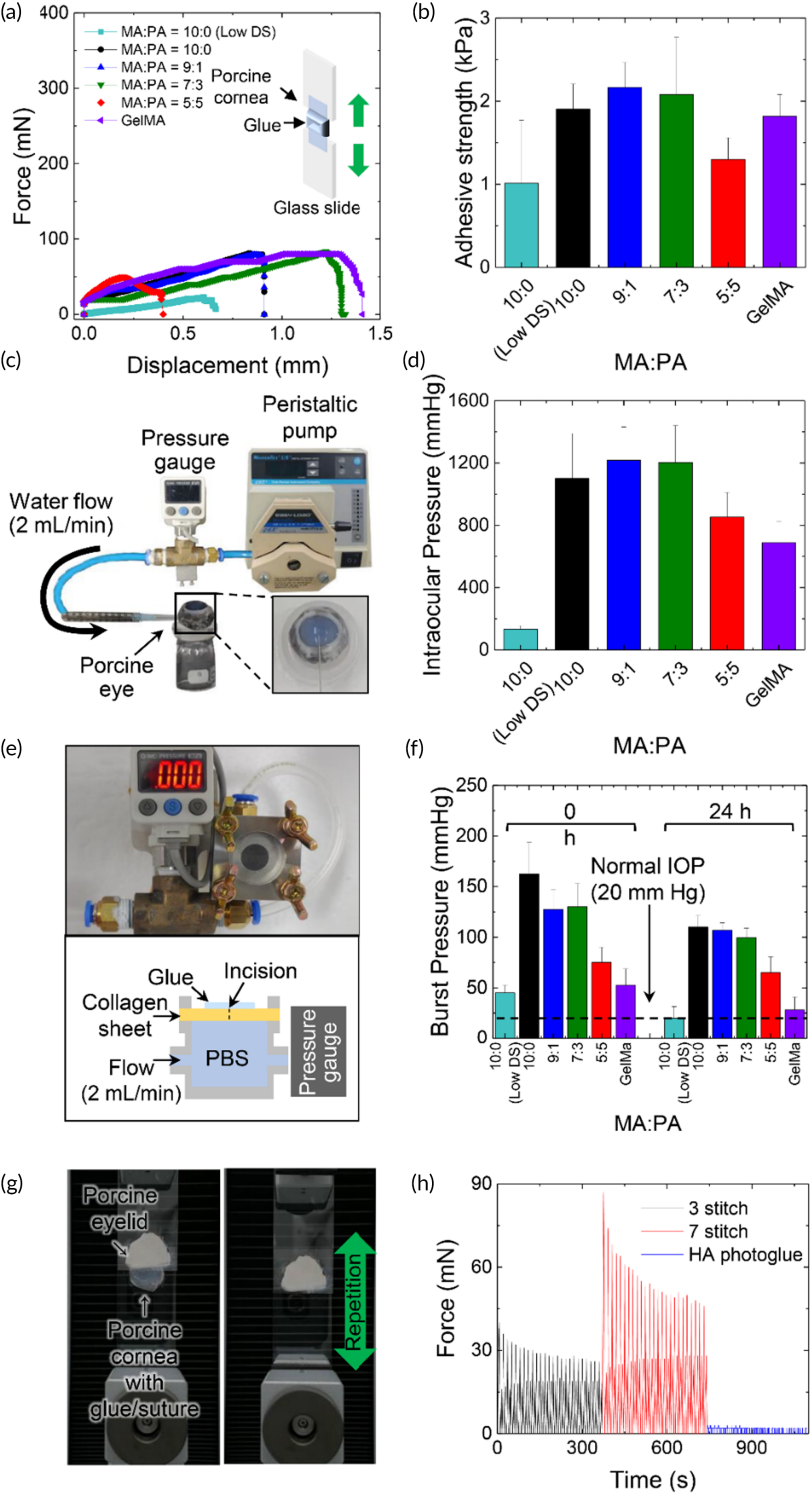
(a) Representative profiles of wound closure tests to measure the adhesion of photoglues bonded between the corneal tissues. (b) Adhesive strength of photocured glues (*n* = 5) (c) Illustration of the experimental set‐up for the burst pressure test of HA photoglues and MA‐functionalized gelatin (GelMA) photoglue attached on the porcine eye. (d) Burst pressure of photocured glues (*n* = 5) (e) Experimental set‐up of the burst pressure test using a collagen sheet. (f) Burst pressure of photocured glues after photocuring and 24 h immersion in water (*n* = 5) (g) Schematic illustration for the friction test using a porcine cornea against porcine eyelid, mimicking eye blinking. (h) Frictional force profiles obtained from HA photoglues and sutures during the repetitive eyelid motion. Values and error bars represent the mean and the standard deviation

To use HA photoglues as an ocular tissue adhesive, it should provide a firm adhesion to the wet mucosal surface withstanding IOP and maintain structural stability with minimal ocular sensation during the wound healing process. The adhesive properties of the HA photoglues with different MA:PA ratios were evaluated through wound closure testing using porcine corneal tissue, as illustrated in Figure 5a. Representative force‐displacement curves obtained from the wound closure tests under tensile stress are shown in Figure 5a. A 10% (w/w) GelMA solution with 0.1% (w/v) PI was used as a control. The introduction of PA moieties in HAMA‐PA improved the adhesion strength of HA photoglues (compared to HAMA), but the high substitution rate (>50%) of PA in HAMA‐PA reduced the adhesive performance (Figure 5b). Interestingly, HAMA‐PA (7:3) glues maintained tissue adhesion following an elongation of ~1.4 mm, highlighting its superior resistance against mechanical stress compared to HAMA with a short photo‐crosslinkable MA group (Figure 5a).

As the leak of aqueous humor from the anterior chamber of the eye can occur during ocular injury or surgery, a sealing property withstanding IOP is required for an ocular adhesive. Burst pressure tests were performed to investigate sealing capacity. For this test, a 5‐mm full‐thickness incision was created using a 2.8 mm keratome on the cornea in the explanted porcine eyeball and then sealed by in situ photo‐crosslinking with either HA photoglues or GelMA glue (as control). The sealed cornea was fixed in a burst pressure test chamber connected to a peristaltic pump and a pressure gauge with an 18G syringe needle. Water was continuously injected at a flow rate of 2 ml min^−1^ to increase the pressure until the applied glues burst (Figure 5c). The burst pressures of explanted eyeball sealed with HA photoglues prepared using HAMA‐PA (9:1) and HAMA‐PA (7:3) were approximately 1200 mmHg, which is 80% higher than that of the GelMA group (700 mmHg) (Figure 5d). Even in burst tests using punctured thin collagen sheets (Figure 5e), the HA photoglues showed sufficient burst pressure (~120 mmHg) to withstand normal IOP (20 mmHg) in the human eye.^37^ Notably, the burst pressure of the HA photoglues decreased by 10–20% after dipping in PBS for 24 h, highlighting the structural stability of HA photoglues with a high degree of substitution of photo‐crosslinkable groups (Figure 5f).

In dissolution tests in a wet environment at 37°C (Figure S8), the HA photoglues exhibited a negligible weight loss of less than 10% following initial swelling for 24 h while the weight of photocured GelMA glues gradually decreased after immersion in PBS at 37°C, possibly because of the dissolution of uncrosslinked gelatin with a sol–gel transition temperature of approximately 30°C.^38^ Considering the superior mechanical adhesive properties, HAMA‐PA (7:3) was selected as the photo‐crosslinkable polymer used in ocular glues for further study.

Since the eye blinks approximately 20 times per min,^39^ mechanical irritation by the adhesive attached to the ocular surface should be minimized during repetitive blinking. To investigate the mechanical stimulation caused by the HA photoglue attached to the corneal surface upon blinking, we measured the frictional force generated by the HA photoglue during 100 cycles of artificial blinking motion (Figure 5g). Sutures were used as controls because they are the gold standard for adhesion or sealing of the corneal tissue. As the suture stitch increased (3–7), the average frictional force increased from 31.37 ± 4.17 mN to 62.5 ± 12.58 mN. Interestingly, the HA photoglue exhibited negligible mechanical stimulation, resulting in a friction force of 2.40 ± 0.66 mN (Figure 5h). Cyanoacrylate glues commonly used in ophthalmologic surgery showed the average frictional force of 329 ± 23.28 mN (Figure S9). The lubricating properties of HA would be beneficial in minimizing ocular foreign body sensation and mechanical stimulus‐induced inflammation associated with the material of the adhesive glue by eye blinking.^40^

### 3.5 Optical properties of HA photoglues

**FIGURE 6:**
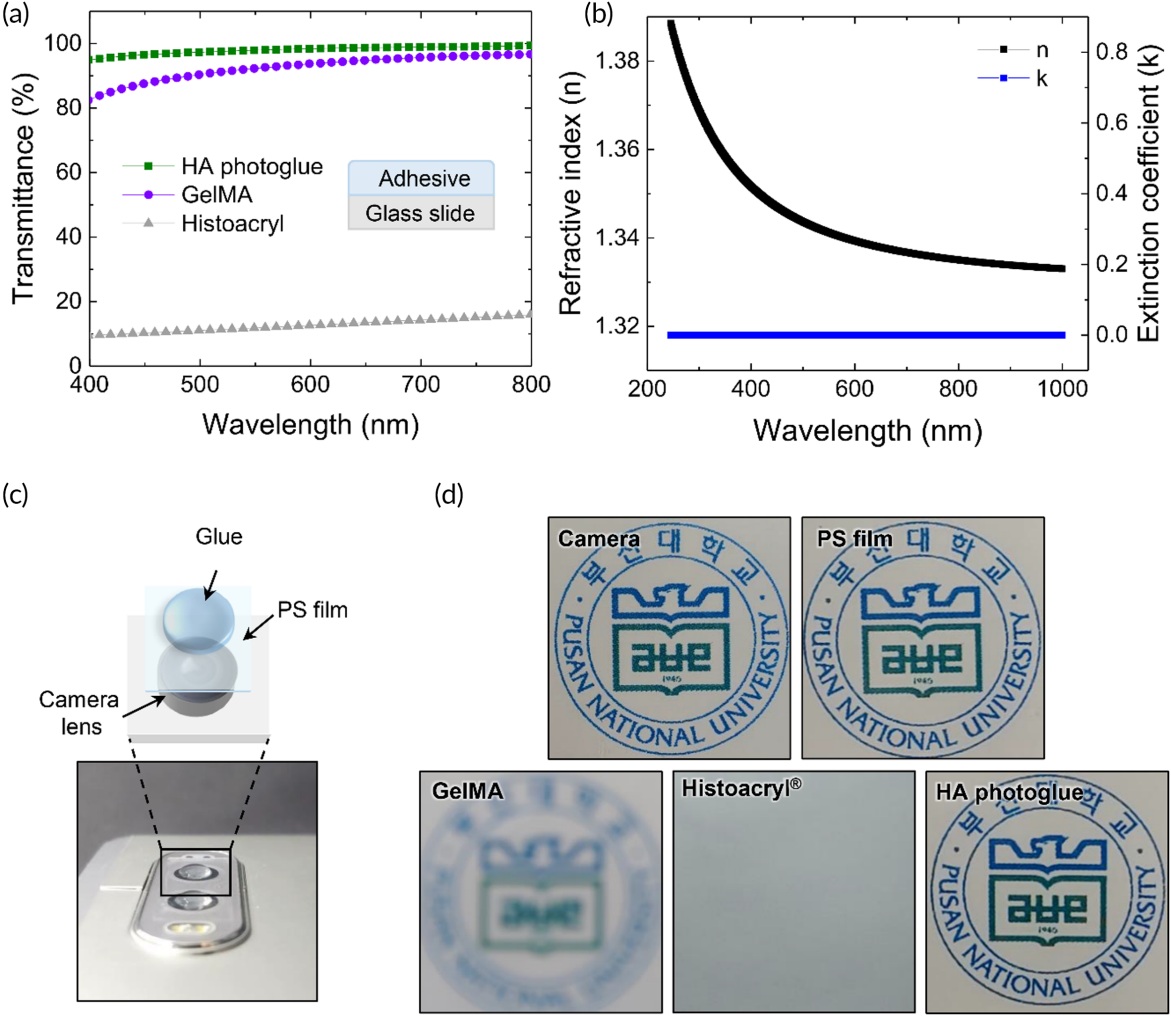
(a) Light transmittance of photocured glues and Histoacryl® films. (b) Refractive index (*n*) and extinction coefficient (k) of a HAMA‐PA (7:3) hydrogel prepared after photocuring. (c,d) Visual tests through Histoacryl® films and HAMA‐PA hydrogel films using a smartphone camera. (c) Experimental set‐up for visual tests. (d) Photographs taken through the photocured glue layer on polystyrene (PS) substrates attached on a camera lens. Camera phone image obtained before or after attaching the PS films

The cornea is the transparent front part of the eye, allowing light to enter the eye for vision. Thus, ocular adhesives must be transparent and have similar optical parameters, such as the refractive index, with corneal tissue. Figure 6a shows the transmittance of light in the visible region (400–800 nm) through photocured glues and conventional cyanoacrylate tissue glue (Histoacryl®). Although Histoacryl® is not approved for ocular use, it is widely used in clinics when corneal sealing is urgently needed.^41^

The HA hydrogel layers prepared by photo‐crosslinking of HA photoglues for 4.3 s exhibited excellent transparency, resulting in a light transmittance of over 95% over the entire visible range (Figure 6a). In the case of the photocured GelMA hydrogel, the light transmission was measured to be 80–90% in the visible range. Histoacryl® glue, consisting of *n*‐butyl‐2‐cyanoacrylate, provided fast‐curing kinetics, but after curing, it formed an opaque film, allowing only 10–20% of the light to pass through the glue layer (Figure 6a). Interestingly, the HA photoglue showed similar refractive index and extinction coefficient to those of human cornea (Figure 6b).^42^ The optical properties of the HA photoglue were not affected after further swelling by absorbing water in the simulated eye environment (Figure S10). To assess the effect of photocured glues adhered to the ocular surface on vision, a photograph was taken through the glue layer formed on a transparent polystyrene (PS) film (Figure 6c). The photograph taken through the GelMA‐coated film produced an unclear image, and when the film was coated with Histoacryl® glue, it was difficult to recognize objects due to a blurry image. The HA photoglue on the film was not affected by the image quality, showing optical performance similar to that of a camera lens (Figure 6d). This vision test clearly demonstrates the benefits of HA photoglues as a tissue adhesive material, especially for ocular applications.

### 3.6 Efficacy and safety of HA photoglues in a rabbit corneal incision model

**FIGURE 7:**
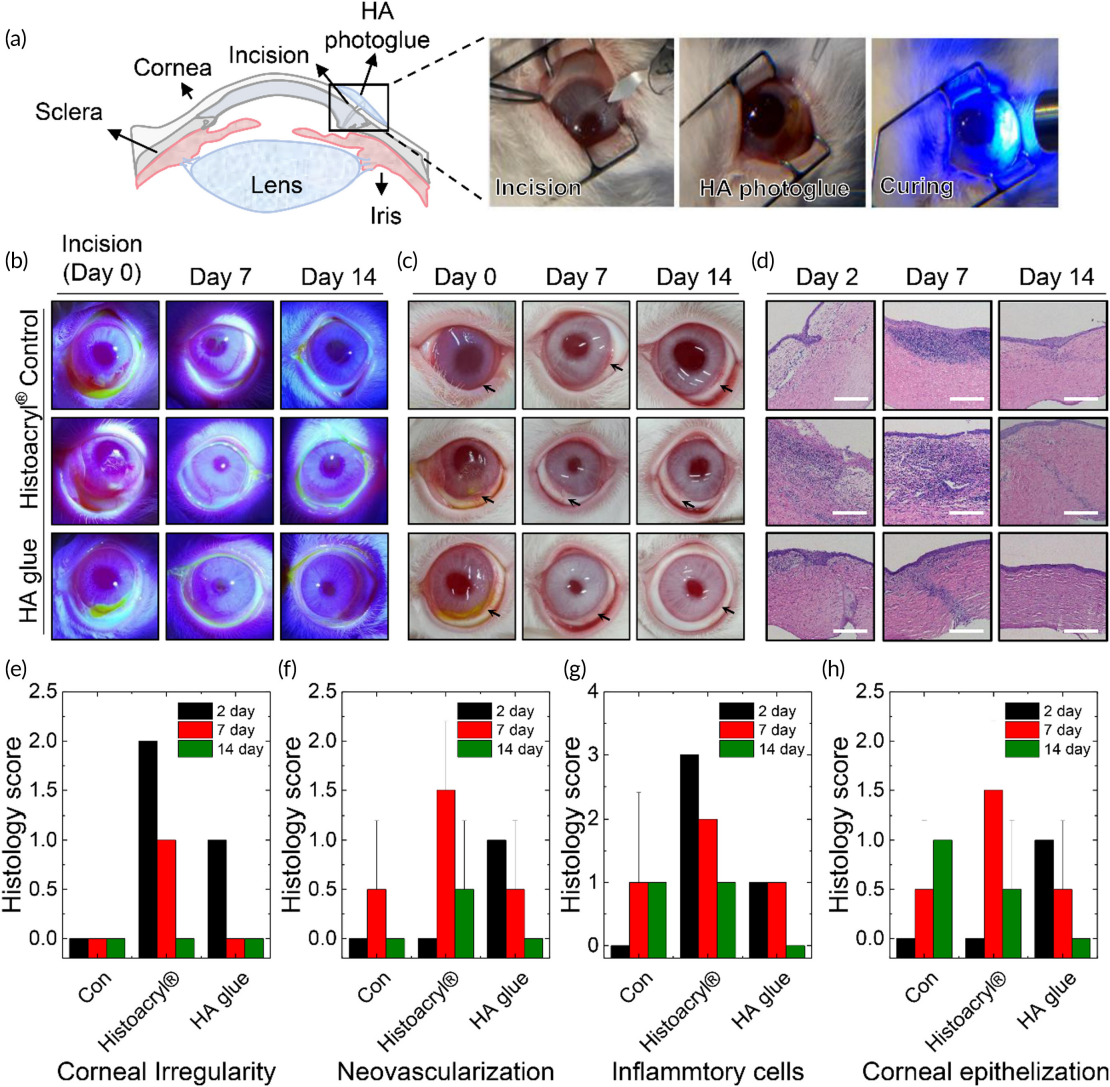
(a) Illustration and photographs showing the experimental procedures for sealing of rabbit corneal incision using HA photoglues; Incision of cornea with 2.8‐mm keratome (leakage from anterior chamber), application of the HA glue on the incision site, and photocuring by light exposure for 4.3 s to seal the incision. (b) Representative photographs showing the Seidel tests at different time points. No treatment group after incision was used as a control. (c) Photographs for visual inspection. The incision site is indicated by the arrow. (d) Histological images of explanted rabbit eye after hematoxylin and eosin (H&E) staining at different time points. (Scale bar: 250 μm). (e‐h) Histology score for (e) corneal irregularity, (f) neovascularization, (g) infiltration of inflammatory cells, and (h) corneal epithelization obtained from histological examination of the tissue shown in (d) (*n* = 3). The score is indicated as follows: 0 (Negligible), 1 (Mild), 2 (Moderate), and 3 (Severe). Values and error bars represent the mean and the standard deviation

We next used experimental animals to examine the *in vivo* wound healing efficacy of the HA photoglue and its biological safety regarding an inflammatory response in a rabbit corneal incision model. After creating a 5‐mm corneal incision on a rabbit eye causing anterior leakage with a 2.8‐mm keratome, 20 μl of HA photoglue was applied to the incision site and photo‐crosslinked by light exposure for 4.3 s (Figure 7a, see Video S1). Commercial cyanoacrylate‐based glue (Histoacryl®) of 20 μl was used as a control. The Histoacryl® glue solidified quickly upon contact with the wet corneal surface.

Ocular sealing with tissue glues following injury was assessed using the Seidel test, which examines the presence of aqueous humor leakage from anterior chamber of the eye. A fluorescein strip containing 10% fluorescein is applied topically to the affected cornea, and examined with a cobalt blue filter. As shown in Figure 7b, the positive Seidel test showed leaking the fluorescein due to dilution of fluorescein caused by the aqueous leakage in the cornea (0 day in the control group). However, after applying the glue as a protective seal, no leakage of aqueous humor from the anterior chamber was observed (0 day in glue groups). The glue layer in both groups maintained its adhesion to the cornea for 1 week, and then fell off during the re‐epithelialization process in wound healing (14 days), as observed previously.^43^

The efficacy and safety of the ocular glues applied to the incision site of the cornea (marked by arrows) were investigated by visual inspection and histological analysis. The HA photoglue following photocuring formed a transparent and smooth surface on the injured cornea. However, Histoacryl® glue formed an opaque and rough layer on the corneal surface (0 day in Figure 7c). When Histoacryl® glue flowed to the eye rim before curing, it resulted in adherence between the cornea and the eyelid, requiring additional surgery to separate tissues (3/3) (Figure S11A). One week after incision, ocular hyperemia was observed in the control group (3/3) and Histoacryl® groups (3/3). Furthermore, synechiae related with inflammation after corneal incision were observed in the control group (2/3) (Figure S11).

In histological evaluation (Figure 7d), the HA photoglue showed uniform corneal epithelial regeneration with minimal inflammation in the early stage (Day 2), whereas the Histoacryl® group formed an irregular layer with marked infiltration of inflammatory cells like polymorphonuclear cell and macrophage. On Day 7, the control and Histoacryl® groups exhibited an abnormal wound healing process of epithelial tissue, possibly due to inflammation of the corneal tissue induced by improper sealing. In contrast, the regenerative epithelial layer was observed in the tissue sealed with HA, indicating a wound healing process. Although epithelialization was completed in all groups at 2 weeks after incision, the HA photoglue group presented relatively uniform epithelial and stromal layers in the cornea compared to the other groups. Based on histological examination (Figure 7e–h), a significant difference existed between groups for irregularities in corneal layers, neovascularization, infiltration of inflammatory cells, and corneal epithelization, especially after postoperative Day 14. This result indicated the HA photoglue group had a lower incidence among all groups and thus improved healing efficacy.

## 4 DISCUSSION

The fast‐curable hydrogel‐forming photoglue based on multilength photo‐crosslinkable HAs suggests a new strategy for developing tissue‐specific adhesives with high potency for translation into clinics. By introducing multiple photo‐crosslinkable groups of different lengths into HA polymer chains in a controlled manner, the mechanical and adhesive properties can be manipulated by regulating the internal structure of the crosslinked hydrogel network. In particular, the multilength network structure in HA photoglues can be attributed to the effective dissipation of mechanical stress in the short chain, while the relatively longer chain delays the rupture process and consequently increases the elongation.^44^ In addition, the single‐component glue prefilled in syringes would be advantageous, compared to multi‐component glues using reactive chemical gelation, for fast application without a mixing procedure.

HA is selected as an ideal ocular adhesive material because it is a biodegradable biopolymer present in the vitreous humor of the eye and approved by the FDA for use as a vitreous replacement during eye surgery.^45^, ^46^ In addition, HA promotes cell migration and proliferation, thereby facilitating rapid repair of injured corneal wounds.^47^, ^48^ Aside from the safety and biological function of HA, it possesses a suitable chemical structure to functionalize effective photo‐crosslinking. The four OH groups in one repeating unit of HA enable a high degree of substitution of photo‐crosslinkable groups, so that the HA photoglue presented in Figure 4 can achieve gelation in 2.5 s under a total light dose of ~0.57 J cm^−2^.

**FIGURE 8:**
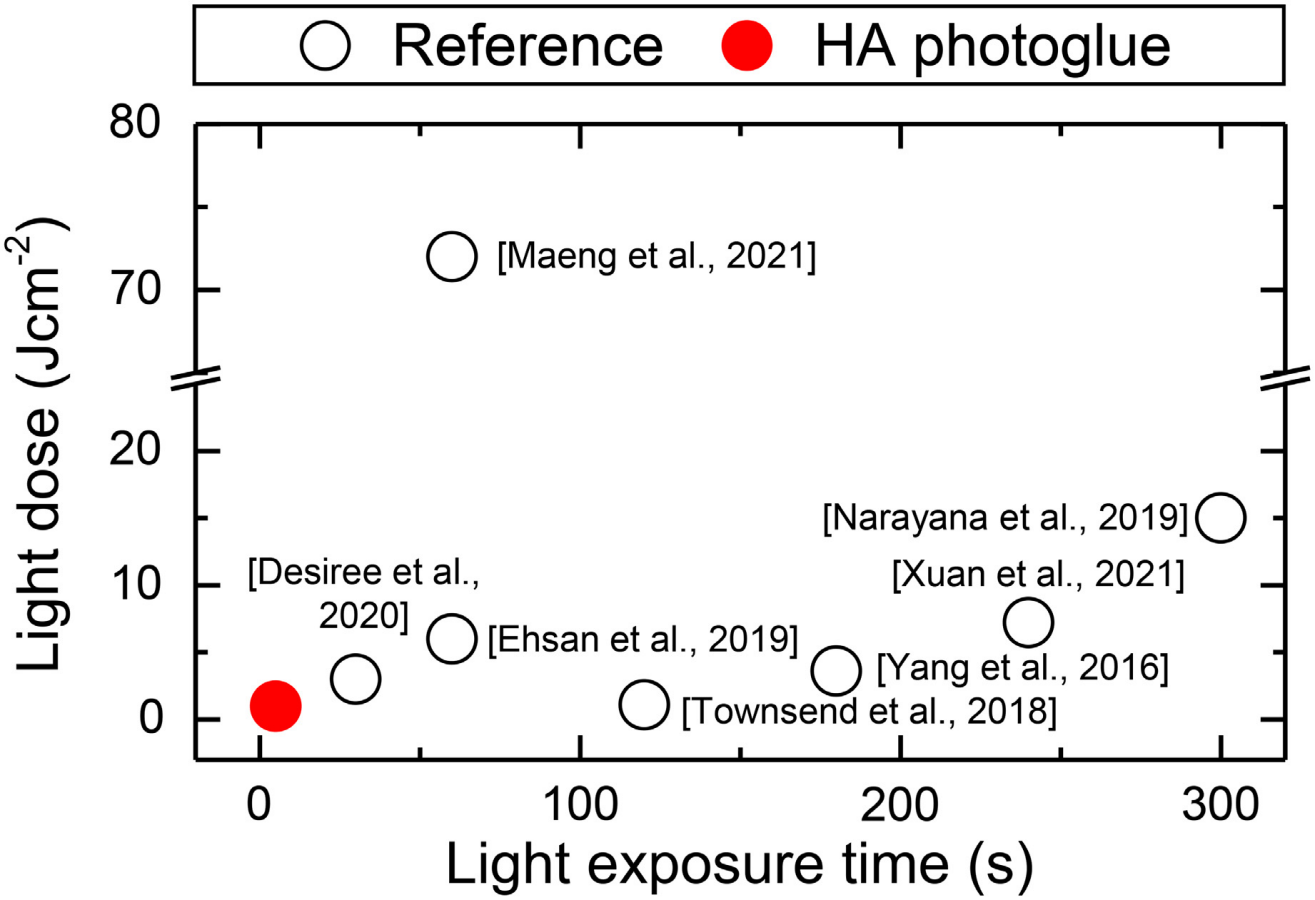
Comparison of the curing time and light dose between previously established photo‐crosslinkable polymers (black open circles) and the HA photoglues (red closed circles) described in this study

As shown in Figure 8, compared with previously reported photocuring conditions for tissue adhesives, the HA photoglue requires very short and low light doses (less than 5 s and 1 J cm^−2^) to produce a firm tissue adhesion with good mechanical stability. Given the high molar extinction coefficient (254 M^−1^ cm^−1^ at 365 nm) in the UV–Vis region of PI (LAP) used in this study (Figure S12), it is expected to achieve sufficient adhesion with a lower energy of 0.3 J cm^−2^ or less if a single wavelength light source of 365 nm is used. Considering the high standard of light safety requirements, this fast and safe photocuring system would be critical for the translation of HA photoglue into the clinic.

## 5 CONCLUSION

In this work, we demonstrated a new tissue glue that provides instant adherence to target wet tissue activated by low‐energy light exposure and suitable mechanical and adhesive properties for applications of ocular surface injury. For the first time, we designed and synthesized copolymeric HAs with multilength photo‐crosslinkable groups through one‐step process, enabling the control of crosslink lengths in photocured hydrogels, thus, improving mechanical and adhesive properties of tissue glues. Depending on the substitution ratios of two different photo‐crosslinkable moieties (forming multiple crosslink lengths) in HA polymeric chains, the mechanical properties can be easily tuned to attain tissue‐specific properties. This approach offers a straightforward and reproducible method to modulate the material properties of hydrogels with multilength networks. By achieving a high total substitution rate (more than 200%) of photo‐crosslinkable groups through a one‐step synthesis process, HA photoglues can be firmly crosslinked in less than 5 s under cytocompatible curing conditions that use a minimal PI amount and meet light exposure safety guidelines. In addition, the photocured HA hydrogel adhesive provided a clear vision with similar ocular parameters, such as refractive index with the human cornea and a lubricating surface with minimal mechanical sensation. By using HA as an adhesive material, this photoactive glue showed enhanced corneal regeneration in an *in vivo* rabbit incision model compared to the cyanoacrylate surgical glue (Histoacryl®), which is commonly used in ophthalmology. Since the drug loading and release kinetics from the hydrogel adhesive can be easily controlled by the formulation of HA photoglues, this on‐demand instant tissue adhesive adhered to wound sites can serve as a long‐term and effective drug delivery reservoir to enhance drug permeation into the eye.

## CONFLICT OF INTEREST

Hyeseon Lee, Samdae Park and Seung Yun Yang are inventors of patent filed by Pusan National University and SNvia. They have a potential financial interest in a company (SNvia) developing surgical glue products.

## AUTHOR CONTRIBUTIONS

**Hyeseon Lee:** Conceptualization (equal); investigation (equal); writing – original draft (equal). **Ajeesh Chandrasekharan:** Conceptualization (equal); investigation (equal); writing – original draft (equal). **Keum‐Yong Seong:** Investigation (equal); methodology (equal). **Yeon Ji Jo:** Investigation (equal); methodology (equal). **Samdae Park:** Investigation (equal); methodology (equal). **Seonyeong An:** Investigation (equal); methodology (equal). **Seungsoo Lee:** Investigation (equal); methodology (equal). **Hyeji Kim:** Investigation (equal); methodology (equal). **Hyungju Ahn:** Investigation (equal); methodology (equal); writing – review and editing (equal). **Sungbaek Seo:** Writing – review and editing (equal). **Seung Yun Yang:** Conceptualization (equal); supervision (equal); writing – review and editing (equal). **Jong Soo Lee:** Conceptualization (equal); supervision (equal); writing – review and editing (equal).

### PEER REVIEW

The peer review history for this article is available at https://publons.com/publon/10.1002/btm2.10323.

## Supporting information


**Appendix S1** Supporting Information

**Video S1** 

## Data Availability

The data that support the findings of this study are available from the corresponding authors upon reasonable request.
