# Supplementary material for: User‐demand fast‐curable ocular glues enforced by multilength tunable networks
Source: Bioeng Transl Med. 2022 Apr 16;7(3):e10323. doi: 10.1002/btm2.10323 (PMC9472003; doi:10.1002/btm2.10323)
Supplement: Supplementary file 1 — Appendix S1 Supporting Information [file BTM2-7-e10323-s002.docx]

Supporting information

User-demand fast-curable ocular glues reinforced by multilength tunable networks

Hyeseon Lee, Ajeesh Chandrasekharan, Keum-Yong Seong, Yeon Ji Jo, Samdae Park, Seonyeong An, Seungsoo Lee, Hyeji Kim, Hyungju Ahn, Sungbaek Seo, Jong Soo Lee*, and Seung Yun Yang*

Summary sentences

- Hyaluronic acid-based photocurable glues (HA photoglues) achieve firm tissue adhesion under wet and dynamic conditions following non-toxic, low-energy light exposure.
- HA photoglue provides a transparent, watertight hydrogel barrier with a lubricating surface.
- The mechanical and adhesive properties of HA photoglues can be controlled by modulating the crosslink lengths in hydrogels by molecular design.
- HA photoglue exhibits improved wound healing efficacy in a rabbit corneal incision model.

Graphical Table of Contents Image
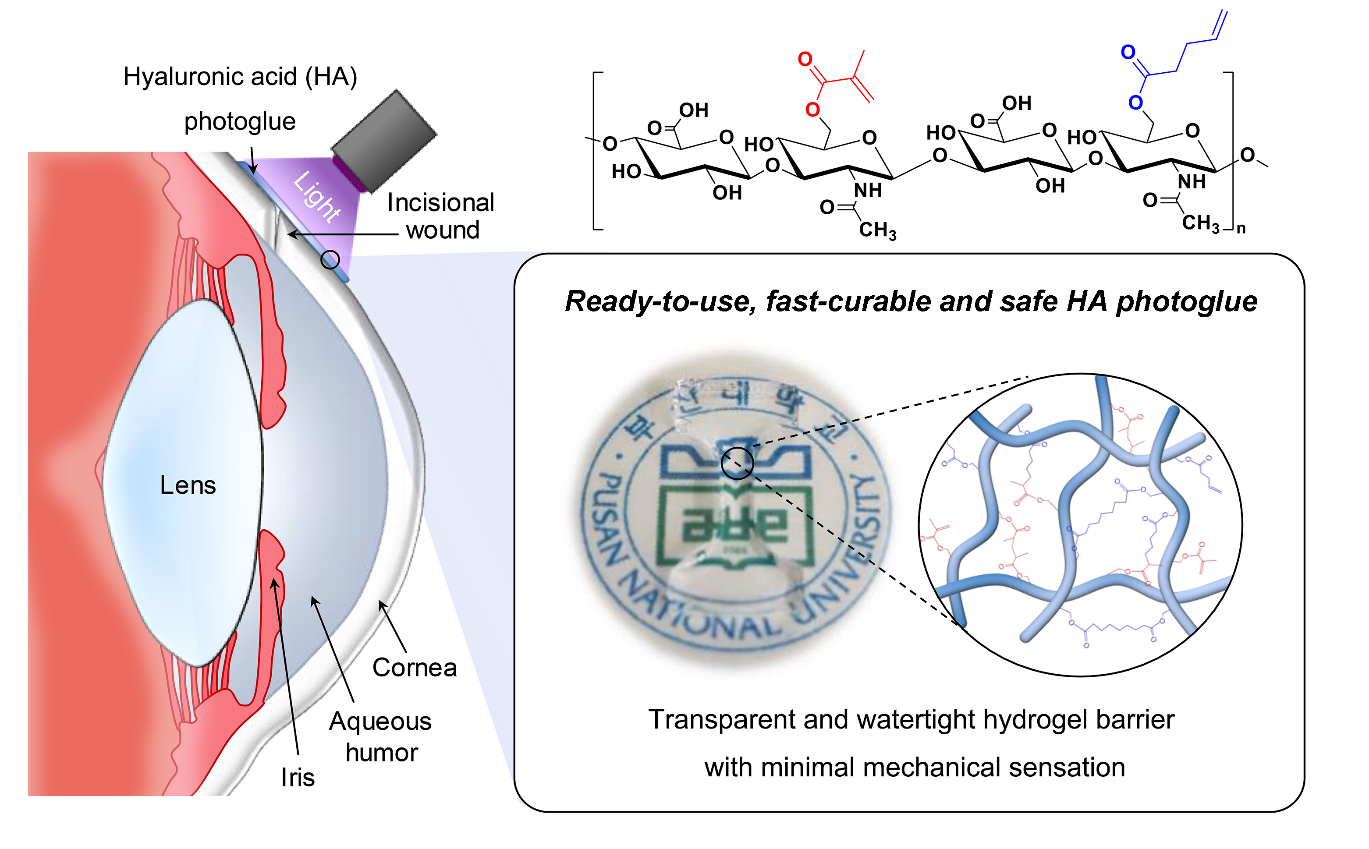


**Table S1.** Molar feed ratios of methacrylic anhydride (MAA):4-pentenoic anhydride (PEA) used for the synthesis of copolymeric hyaluronic acid (HA) with two photocrosslinkable groups (HAMA-PAs) and product ratios of methacrylate (MA) and 4-pentenoate (PA) in the synthesized HAMA-PAs (n = 3).

| **MAA + PEA mole equivalents** | **Feed ratios of MAA:PEA** | **MA:PA** | **Total degree of substitution**  **(MA + PA %)** |
| --- | --- | --- | --- |
| **5** | **5:0** | **10 : 0** | **211** |
| **5** | **4:1** | **8.8 ± 0.08 : 1.2 ± 0.08** | **263 ± 2.5** |
| **5** | **2.5:2.5** | **6.9 ± 0.19 : 3.1 ± 0.19** | **219 ± 24** |
| **5** | **1.5:3.5** | **4.8 ± 0.12 : 5.2 ± 0.12** | **208 ± 10** |

**Table S2.** Correlation length model fit parameters of the small-angle X-ray scattering (SAXS) intensity profiles of the HAMA-PA hydrogels.

| **HAMA-PA hydrogels** | **Correlation lengths (ξ)** |
| --- | --- |
| **MA:PA = 10:0** | **1.67 nm** |
| **MA:PA = 7:3** | **2.05 nm** |
| **MA:PA = 5:5** | **2.22 nm** |


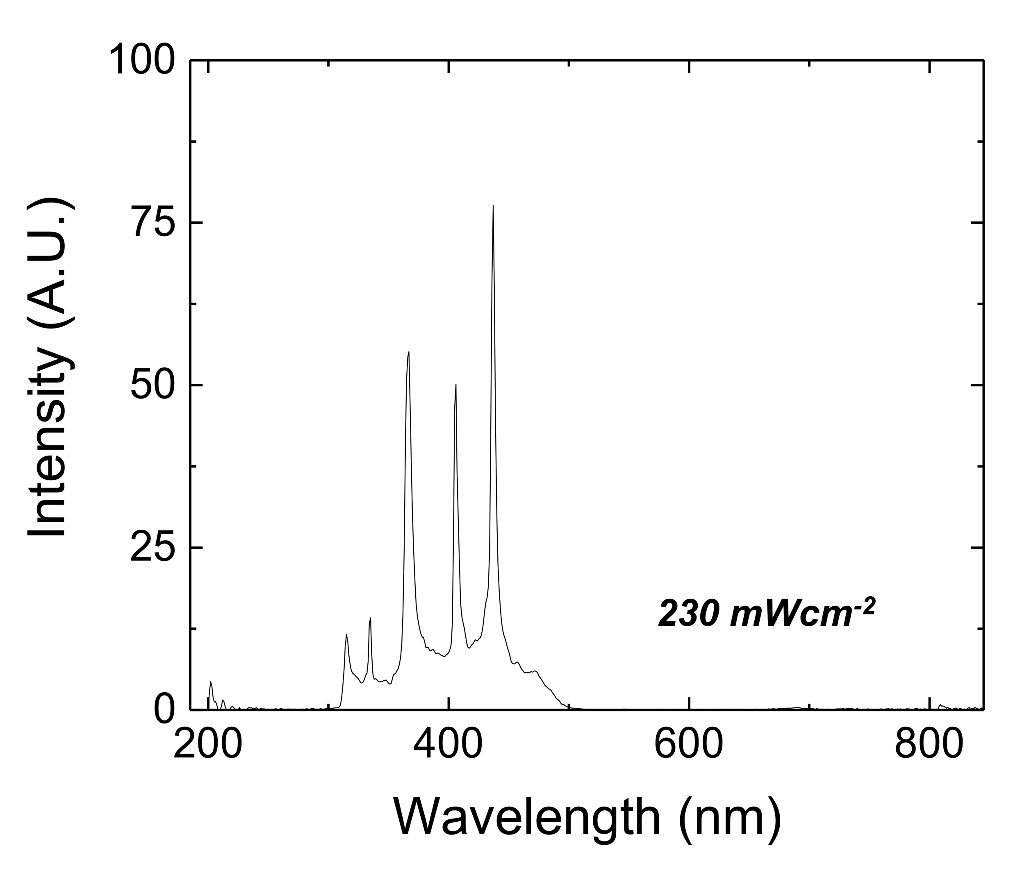


**Fig. S1.** Irradiance and spectrum intensity of the light source (Omnicure s1500) measured by a spectrometer (StellarNet Black-Comet).


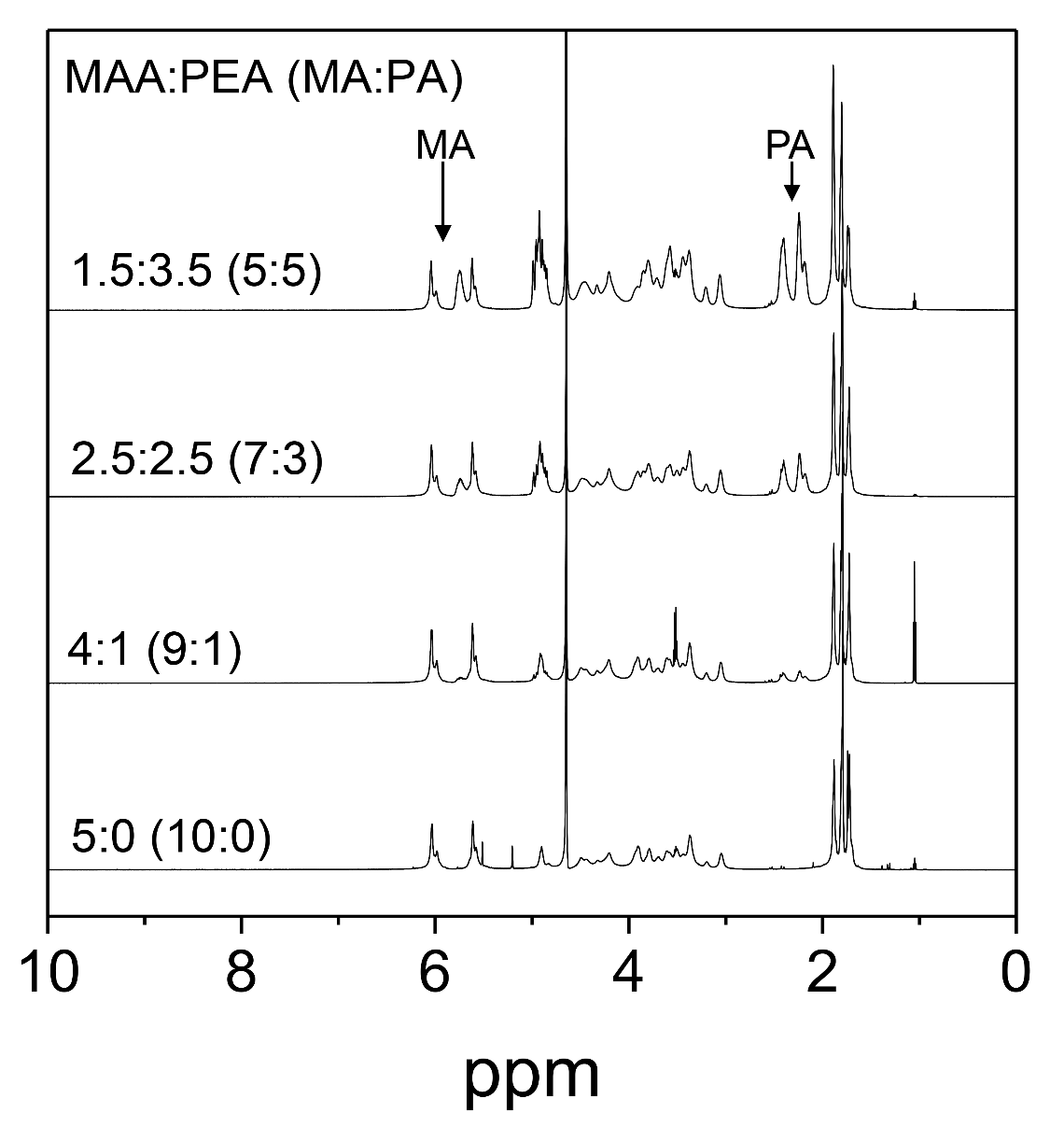


**Fig. S2.** ^1^H NMR spectrum of HAMA-PA prepared by different molar feed ratios of methacrylic anhydride (MAA):4-pentenoic anhydride (PEA). Arrow indicates peaks corresponding to methacrylate (MA) (6.18 ppm) and 4-pentenoate (PA) (2.55 ppm) used for calculating the degree of substitution.


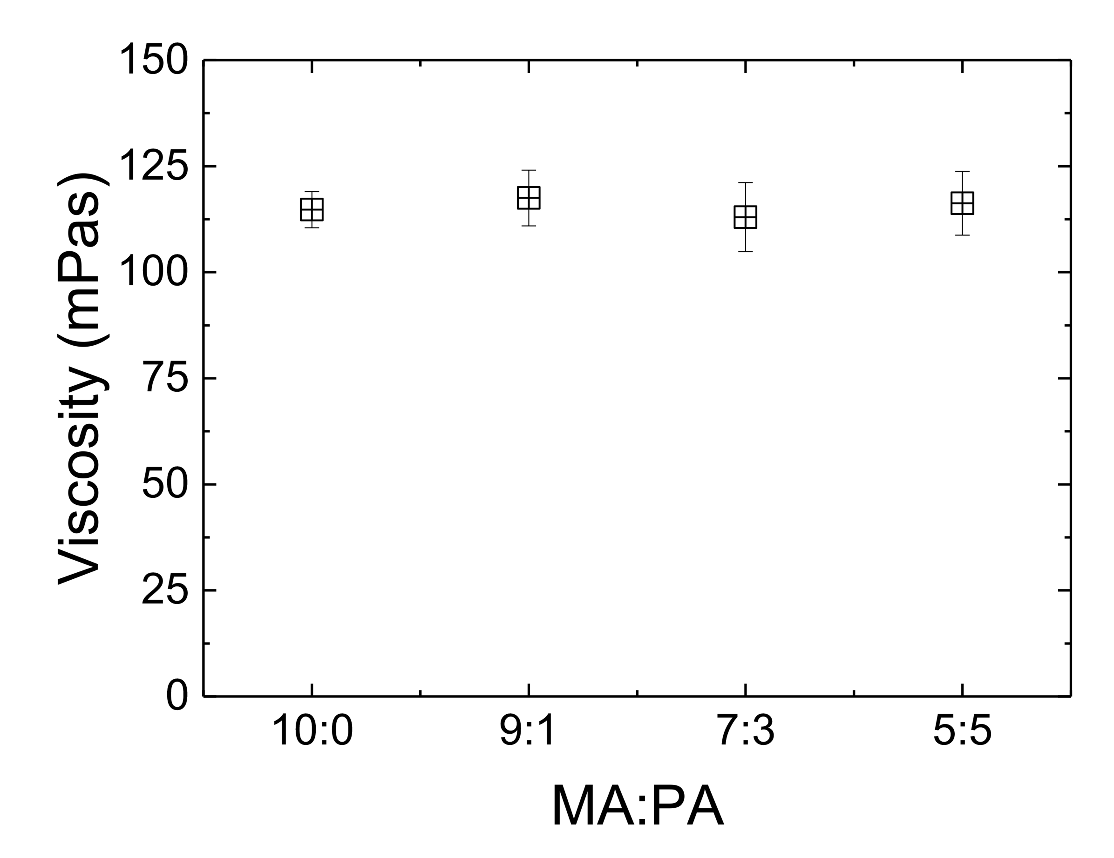


**Fig. S3.** Viscosity of aqueous hyaluronic acid (HA) with two photocrosslinkable groups (HAMA-PA) solutions prepared with different molar ratios between MA and PA in HAMA-PAs.


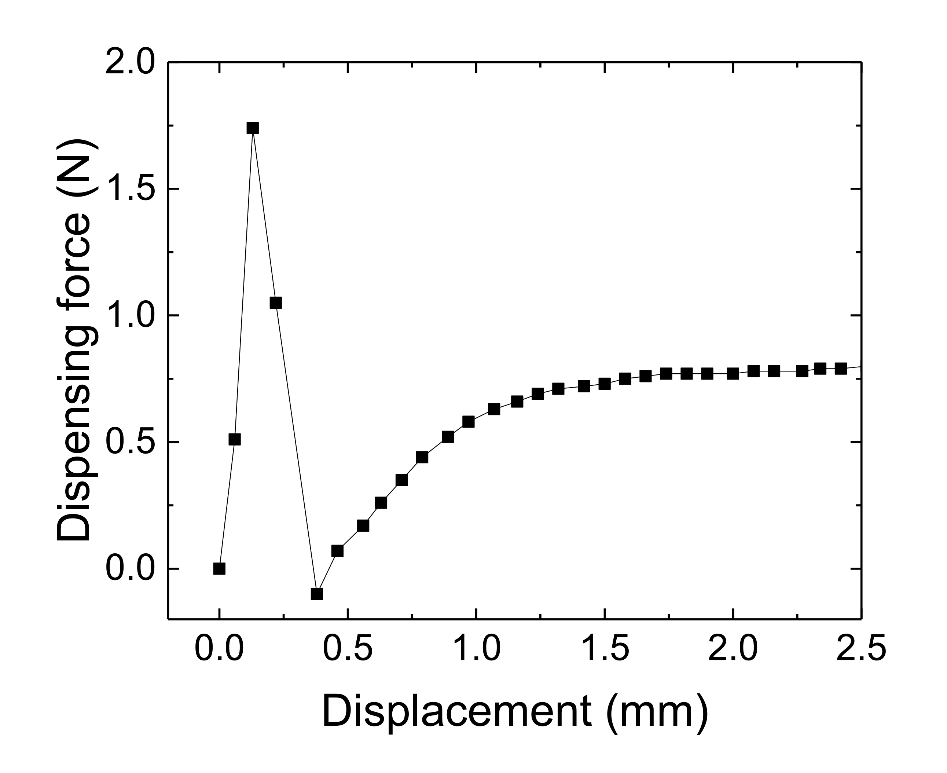


**Fig. S4.** Initial force-displacement profile obtained while compressing the syringe plunger to dispense the 5 wt% HAMA-PA solution.


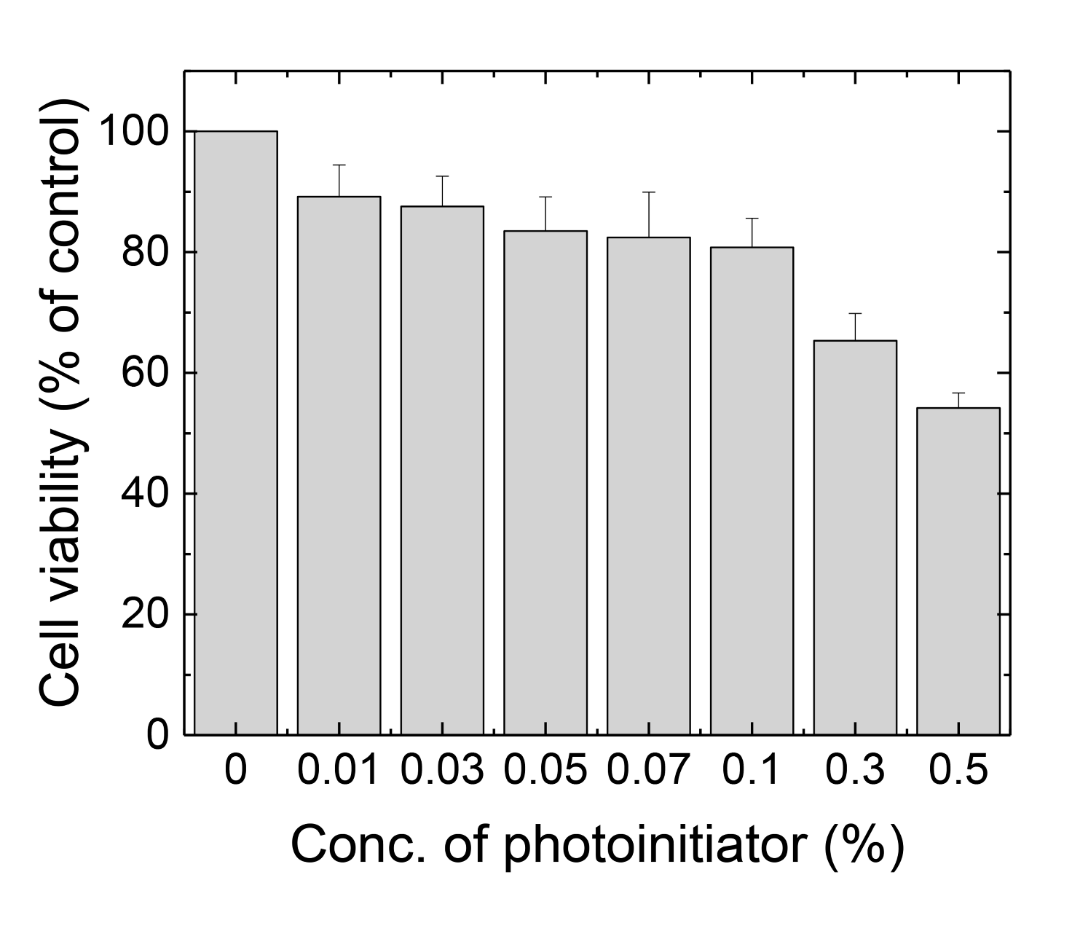


**Fig. S5.** Cytotoxic effects of photoinitiator (PI) concentrations on the rabbit corneal stromal cells (n = 3). The primary corneal stromal cells were isolated from the corneal stroma of New Zealand white rabbit by digestion in sterile DMEM containing 2.0 mg/ml collagenase type L (Gibco, USA) and 0.5 mg/ml hyaluronidase (Gibco) for 1 h at 37 °C. The corneal stromal cells were cultured in DMEM with 10% FBS (Hyclone) and 1% penicillin-streptomycin (Hyclone). To perform the cell viability assay, the cells of passage 6-7 were seeded in 24-well plates at a density of 2×10^4^ cells/well and incubated for 24 h. The experiments were performed with different concentrations of photoinitiator (0.01, 0.03, 0.05, 0.07, 0.1, 0.3, and 0.5% (w/v)) for 24 h. The cytotoxic effect was not significant at a concentration of less than 0.1% PI, showing a cell viability of more than 80% compared to the non-treated group.

**
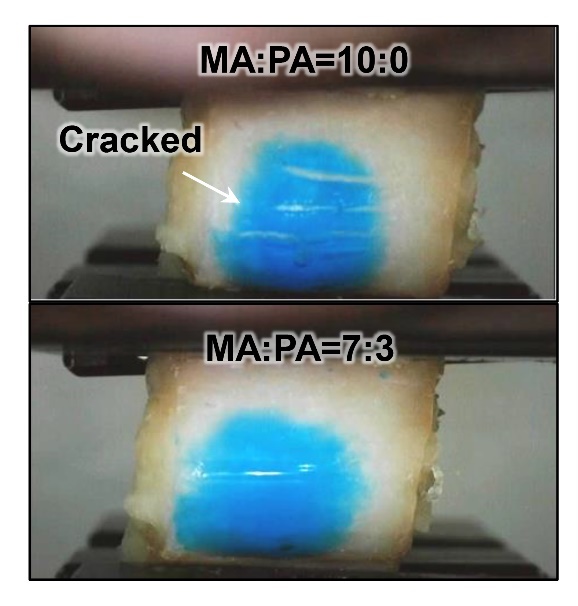
**

**Fig. S6.** Photographs showing the adhesion of photocured HA glues (HAMA and HAMA-PA) on pig skin in bending motion.


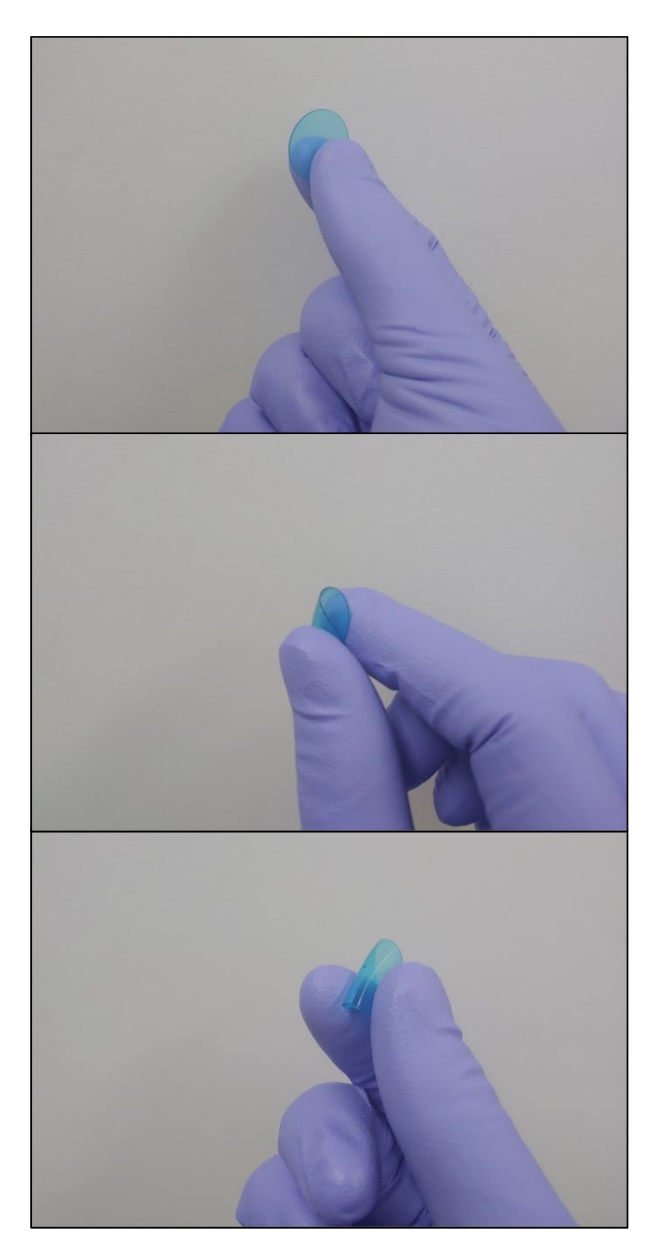


**Fig. S7.** Photographs showing the flexibility of photocured HA hydrogel film (MA:PA 7:3).


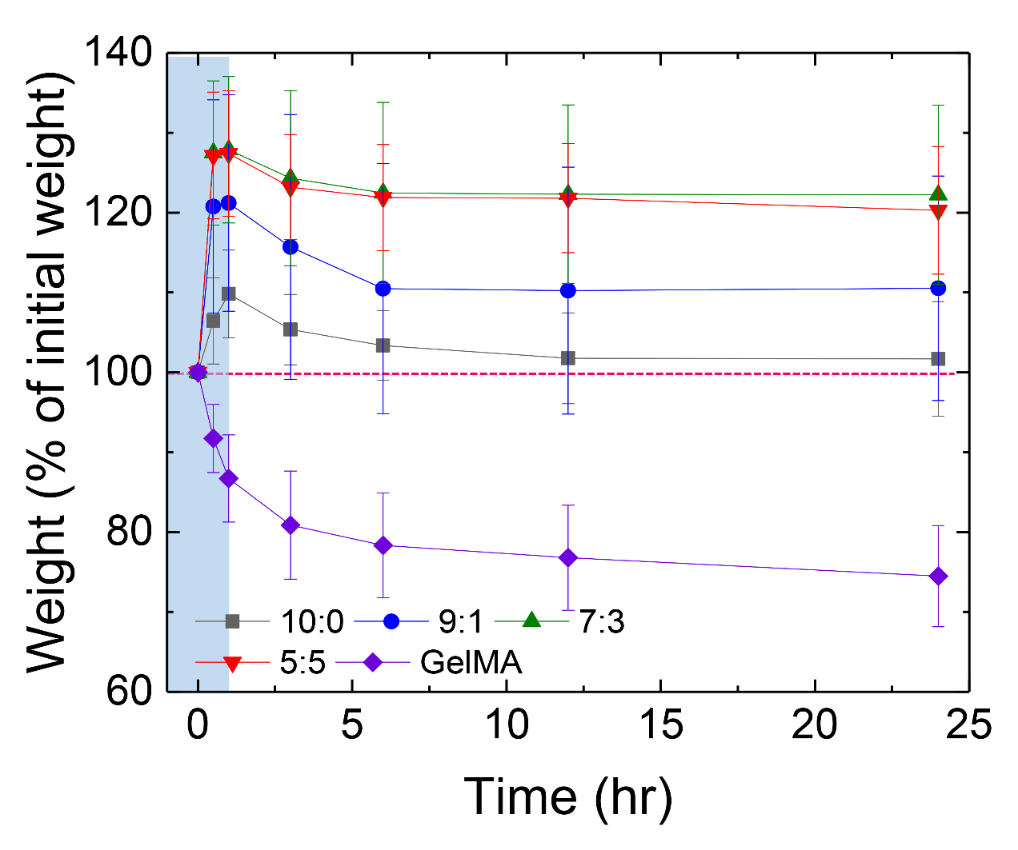


**Fig. S8.** Dissolution test with photocrosslinked hydrogels after immersion in phosphate-buffered saline (PBS) at 37 °C. The HA-based glues exhibited a negligible weight loss less than 10% following the initial swelling for 24 h, while the weight of MA-functionalized gelatin (GelMA) adhesives gradually decreased.

**
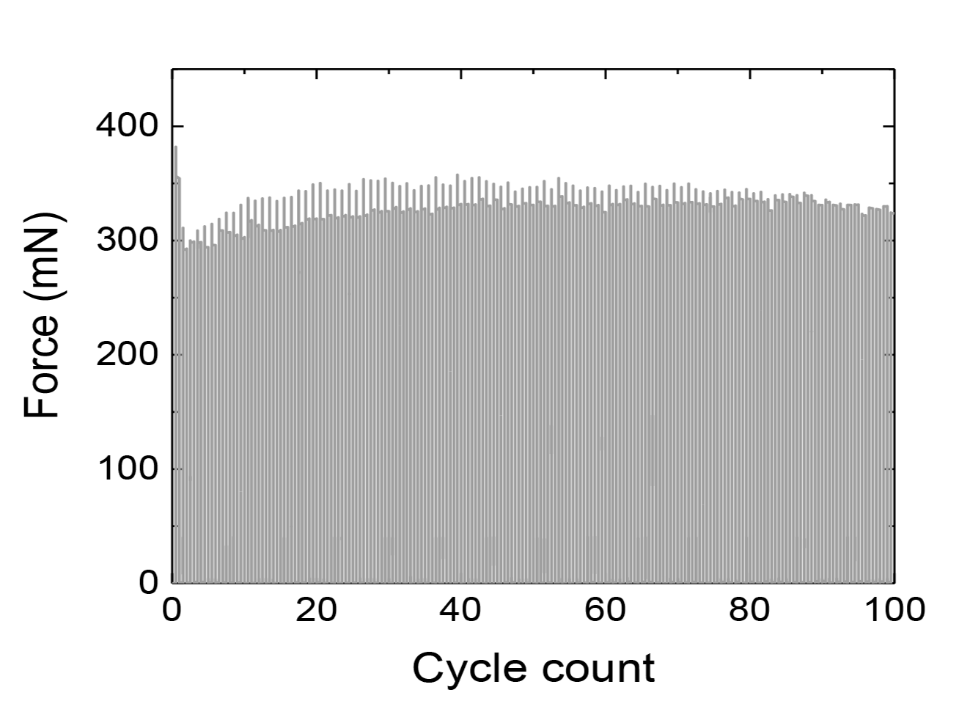
**

**Fig. S9.** Frictional force profiles obtained from Histoacryl® during the repetitive eyelid motion.**
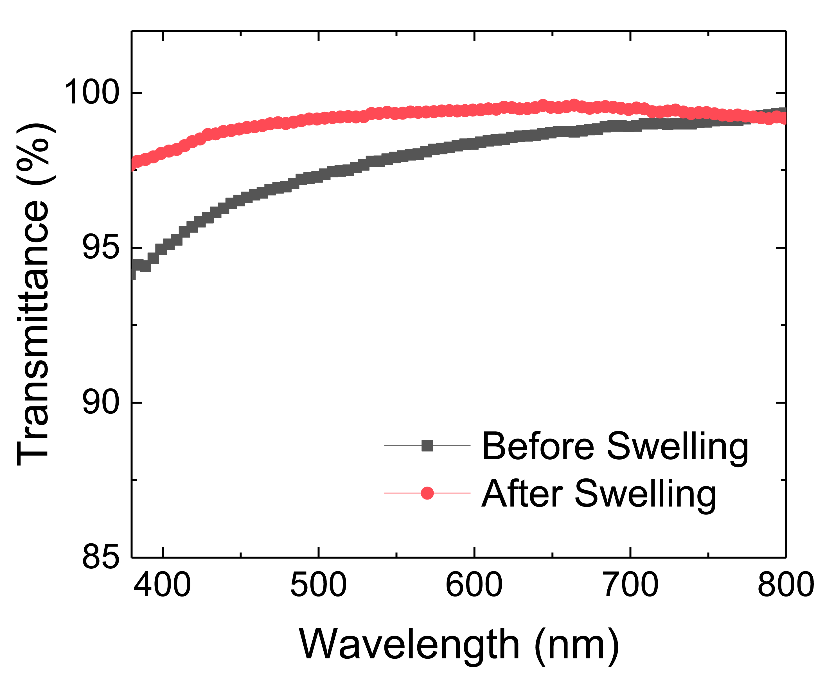
**

**Fig. S10.** Light transmittance of photocured HA hydrogels before and after immersion in PBS for 7 days.


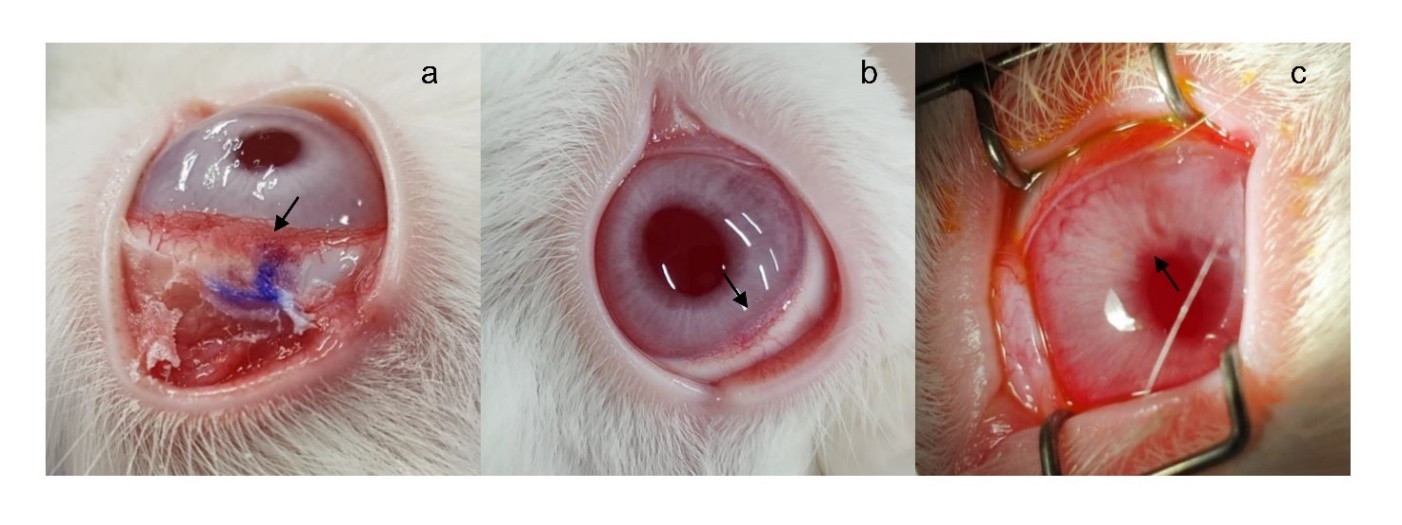


**Fig. S11.** Photographs obtained during visual inspection. (a) When Histoacryl was applied improperly, it resulted in the adherence between the cornea and the eyelid (3/3). (b) Limbal injection and (c) and corneal neovascularization related with inflammation after corneal incision was observed in the control group 1 week after incision.


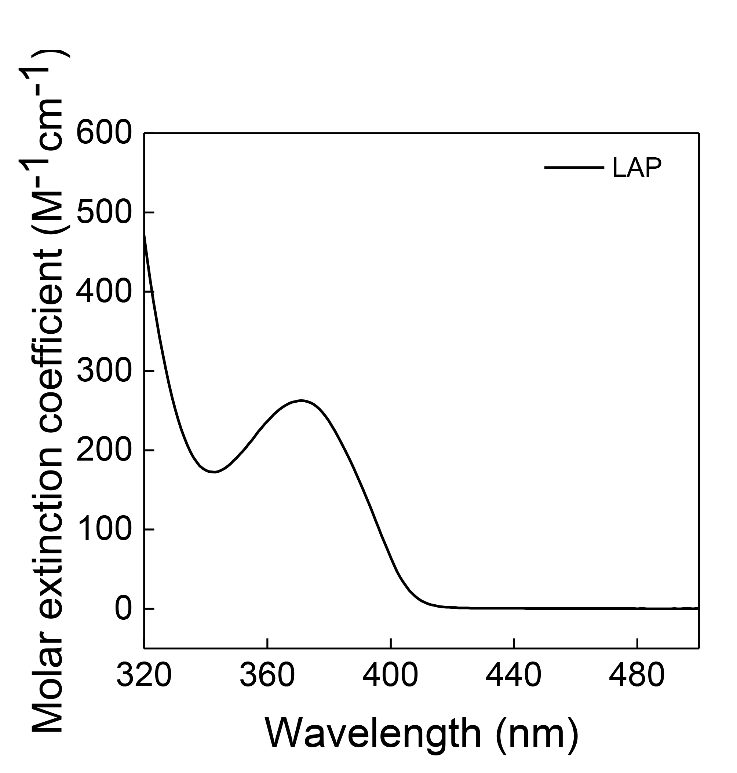


**Fig. S12.** Molar extinction coefficient of the photoinitiator, lithium phenyl-2,4,6-trimethylbenzoylphosphinate (LAP), used in this study.
